# Using the Consolidated Framework for Implementation Research to evaluate a nationwide depression prevention project (ImplementIT) from the perspective of health care workers and implementers: Results on the implementation of digital interventions for farmers

**DOI:** 10.3389/fdgth.2022.1083143

**Published:** 2023-01-23

**Authors:** Johanna Freund, David Daniel Ebert, Janika Thielecke, Lina Braun, Harald Baumeister, Matthias Berking, Ingrid Titzler

**Affiliations:** ^1^ Department of Clinical Psychology and Psychotherapy, Institute of Psychology, Friedrich-Alexander-Universität Erlangen-Nürnberg, Erlangen, Germany; ^2^ TUM Department of Sport and Health Sciences, TU Munich, Munich, Germany; ^3^ Department of Clinical Psychology and Psychotherapy, Institute of Psychology and Education, Ulm University, Ulm, Germany

**Keywords:** implementation, internet-based intervention, telephone coaching, consolidated framework for implementation research (CFIR), prevention, depression, mental health, farmers

## Abstract

**Introduction:**

Depression has a significant impact on individuals and society, which is why preventive measures are important. Farmers represent an occupational group exposed to many risk factors for depression. The potential of guided, tailored internet-based interventions and a personalized telephone coaching is evaluated in a German project of the Social Insurance for Agriculture, Forestry and Horticulture (SVLFG). While user outcomes are promising, not much is known about actual routine care use and implementation of the two digital health interventions. This study evaluates the implementation from the perspective of social insurance employees to understand determinants influencing the uptake and implementation of digital interventions to prevent depression in farmers.

**Methods:**

The data collection and analysis are based on the Consolidated Framework for Implementation Research (CFIR). Health care workers (*n* = 86) and implementers (*n* = 7) completed online surveys and/or participated in focus groups. The surveys consisted of validated questionnaires used in implementation research, adapted items from the CFIR guide or from other CFIR studies. In addition, we used reporting data to map implementation based on selected CFIR constructs.

**Results:**

Within the five CFIR dimensions, many facilitating factors emerged in relation to intervention characteristics (e.g., relative advantage compared to existing services, evidence and quality) and the inner setting of the SVLFG (e.g., tension for change, compatibility with values and existing working processes). In addition, barriers to implementation were identified in relation to the outer setting (patient needs and resources), inner setting (e.g., available resources, access to knowledge and information) and characteristics of individuals (e.g., self-efficacy). With regard to the implementation process, facilitating factors (formal implementation leaders) as well as hindering factors (reflecting and evaluating) were identified.

**Discussion:**

The findings shed light on the implementation of two digital prevention services in an agricultural setting. While both offerings seem to be widely accepted by health care workers, the results also point to revealed barriers and contribute to recommendations for further service implementation. For instance, special attention should be given to “patient needs and resources” by raising awareness of mental health issues among the target population as well as barriers regarding the inner setting.

**Clinical Trial Registration:**

German Clinical Trial Registration: [DRKS00017078]. Registered on 18.04.2019

## 1. Introduction

Depression is a highly prevalent disorder (1) and the leading cause of disability worldwide (2). Yet despite its high burden on individuals and society, depression often remains untreated due to stigma (3) inadequate mental health resources (4), and the preference to manage problems on one’s own (5). Moreover, existing evidence-based treatments for mental disorders can reduce the burden of mental disorders by only one-third (6). Measures to prevent depression are therefore becoming increasingly important (7).

For depression treatment, there are increasing numbers of internet- and mobile-based interventions and research studies (8–12). Internet-based interventions (IBIs) may be widely used, and since they are easily accessible (13), they may help individuals overcome barriers to obtaining face-to-face mental health care (14). According to a recent meta-analyses, IBIs can effectively treat depression (15) as well as subclinical depressive symptoms and help to prevent the onset of major depressive disorder (16). Similarly, tele-based interventions offer many of the same advantages, such as easy access and flexibility (17). The effectiveness of telemedicine services for depression treatment has been demonstrated by a meta-analysis indicating that the outcomes of tele-therapy do not differ from those of face-to-face-therapy (18).

For depression prevention, the potential of digital interventions including guided, tailored IBIs and personalized telephone coaching (TC) is evaluated with several RCTs and implementation studies in the German project “With us in balance” of the Social Insurance for Agriculture, Forestry and Horticulture (SVLFG). Farmers and related occupational groups are exposed to many risk factors for depression such as a high workload, financial worries, poor weather conditions, and health problems (19–23) and have a higher risk of mental health issues, especially depression, compared to other professional groups (22, 24–26). As their help-seeking behavior is often restricted due to its stigmatization (25) and limited mental health care in rural areas (27), the SVLFG introduced digital interventions for their insured members to compliment existing but limited onsite prevention services to overcome barriers to care.

The results of the “With us in balance” project based on a randomized controlled trial (RCT) showed a small effect on the reduction of depressive symptoms through guided IBIs at 9-weeks post-treatment (*d* = −0.28, 95% CI: −0.50 to −0.07) and at 6-month follow-up (*d* = −0.35, 95% CI: −0.57 to −0.14) compared to treatment-as-usual (TAU) (28, 29), while no effects could be found at 12-month-follow-up (28). First results on the effectiveness of the personalized TC demonstrated a small to medium effect on the reduction of depressive symptoms (*d* = −0.39, 95% CI: −0.15 to −0.64) at 6-month posttreatment in comparison to TAU (30). The implementation study “ImplementIT” (31) aims to evaluate the implementation of the digital interventions into routine care. First qualitative results regarding acceptance of and satisfaction with the digital intervention as well as barriers and facilitators for use from the perspective of participating farmers, forest owners, and gardeners have been explored (17, 32, 33). The results indicate that these offers could be suitable for the target group as interviewees reported that digital interventions helped them overcome barriers to treatment and brought specific benefits such as “flexible use”, “anonymity”, and “location independence” (32, 33). At the same time, “time-consuming work life” and “time-consuming private life” were the most often mentioned barriers regarding intervention use (32). However, besides RCT participation, in which low adherence regarding intervention use (28, 29) and recruitment difficulties (34) are reported for IBIs, not much is known about routine care use. Therefore, it is critical to understand the factors that influence the uptake and implementation of both digital health interventions.

As noted in a recent scoping review (35), there is little existing research on the implementation of digital interventions for depression in routine care and leadership and organizational factors have been largely neglected in previous studies on the implementation of digital interventions. Similarly, in the field of depression prevention, there is scant research on the implementation of digital interventions. The existing studies are limited to the internet-based prevention of eating disorders in young adults (36) and depression in adolescents (37). Therefore, further research on the implementation of digital interventions for depression prevention, especially regarding organizational aspects, is needed. In this project, health care workers are involved in the consultation and referral process to digital interventions and thus, play a central role as gatekeepers in the management of depression prevention at the social insurance company. In the broadest sense their working activity is comparable with the referral role of general practitioners (GPs), whose referral behavior can be influenced by skepticism and negative attitudes (38). Therefore, it is essential to understand determinants influencing professionals’ attitudes and behavior. Furthermore, the implementation of the digital interventions leads to a behavioral change among health care workers who previously have not advised on digital health services or health services in general. As recommended in previous studies (39–41), behavioral change should be investigated on a theoretical basis to guide the selection of constructs.

The aim of this study is to evaluate the implementation process from the perspective of health care workers to understand the barriers and facilitating factors in the uptake and implementation of digital interventions to prevent depression in farmers, forest owners, and gardeners. Additionally, the use of dissemination and implementation strategies is examined.

## 2. Methods

### 2.1. Study design

**Figure 1:**
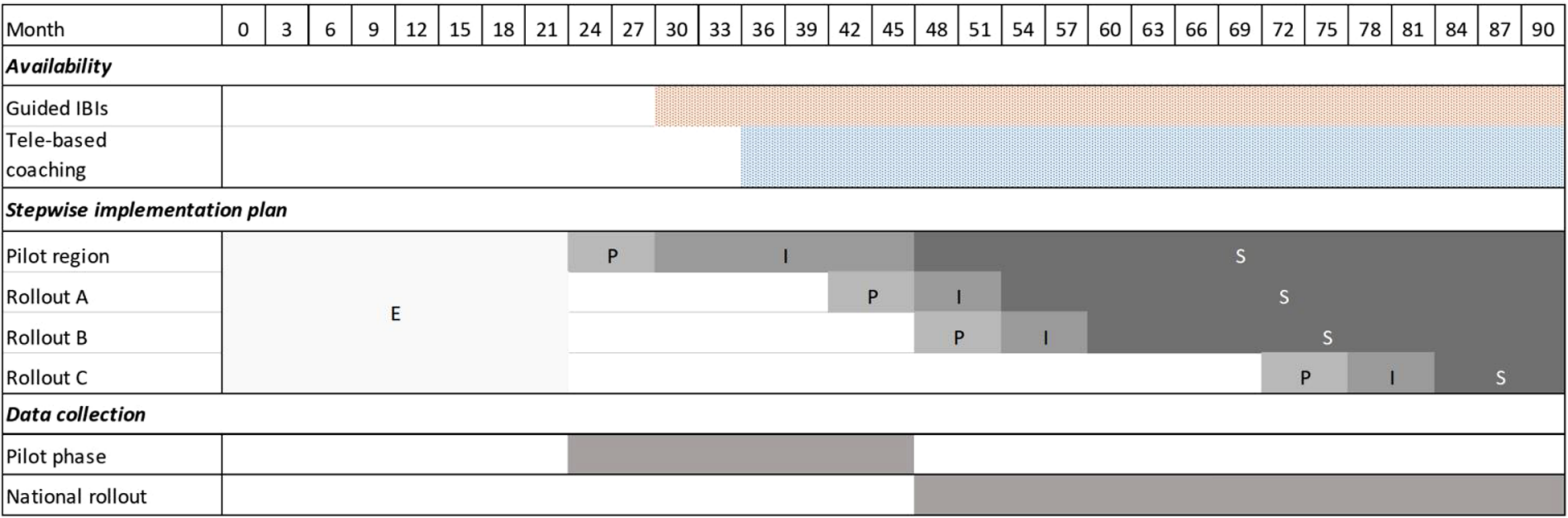
Project timeline including exploration (E), preparation (P), active implementation (I), and sustainment (S) phase per rollout area.

As defined in the study protocol of ImplementIT (31), the implementation study follows a mixed-methods design with quantitative (surveys, reporting data) and qualitative approaches (focus groups, open questions). The stepwise implementation determining the availability of the digital interventions across all federal states in Germany is illustrated in Figure 1. According to the Conceptual Model of Implementation Phases (42), the study can be divided into the phases “exploration”, “preparation”, “implementation” and “maintenance”. The evaluation is based on the established Consolidated Framework for Implementation Research (CFIR) (43), which has already been applied in a similar setting (44). The CFIR (43) unifies constructs from various implementation theories and offers a pragmatic structure to assess complex and interacting states in the implementation across five domains consisting of four to eight constructs. The first domain is related to characteristics of the implemented intervention and includes constructs such as “evidence strength and quality”, “relative advantage”, and “adaptability”. The second domain is the outer setting and includes constructs from the cultural, political, and structural context (e.g., “patient needs and resources”, “external policies and incentives”) that influences the implementation. The third domain includes aspects with regard to the inner setting (e.g., “networks and communications”, “culture”, “readiness for implementation”). The fourth domain is related to characteristics of the individuals involved in the intervention/implementation (e.g., “knowledge and beliefs about the intervention”, “individual stage of change”, “other personal attributes”). The fifth domain is the implementation process and includes constructs such as “planning”, “engaging”, and “reflecting and evaluating”. The study is described alongside Standards for Reporting Implementation Studies; see the StaR checklist (45) as supplementary.

### 2.2. Interventions

The SVLFG advertises its health services through public relation (PR) activities. In addition, insured persons are also advised on the health services by field workers and in-house staff. During consultations at the SVLFG call center, insured people who meet the eligibility criteria of SVLFG receive detailed information on what prevention services they can access. They can choose between digital interventions (e.g., IBIs, TC) or group preventive services delivered onsite. The call center agents carry out the registration process. The digital mental health interventions that are provided free of charge for the insured are described in detail in the study protocol (31) and summarized below.

#### 2.2.1. Guided IBIs

After receiving an access code for the guided IBIs from the SVLFG call center, the participant can register on the service company’s online platform (www.geton-institut.de). The IBIs consist of three intervention phases: (1) the participant completes a *psycho-diagnostic assessment* based on self-report-questionnaires. One of seven trainings is selected based on the assessment and the participant’s individual preferences during the initial phone call with the e-coach. (2) The participant completes the IBI in the *active training phase* with personal e-coach (psychologist who has started at least psychotherapeutic training) guidance consisting of feedback *via* e-mail or phone. The IBI “GET.ON Stress” is based on the transactional model of stress and coping (46), the IBI “GET.ON Chronic Pain” on Acceptance and Commitment Therapy (47), and all other IBIs on Cognitive Behavioral Therapy (CBT) principles (“GET.ON Mood Enhancer”, “GET.ON Mood Enhancer Diabetes”, “GET.ON Recovery”, “GET.ON Panic”, “GET.ON Be smart—Drink less”). The IBIs consist of 6–8 weekly modules including psycho-educative information, exercises, interactive elements (e.g., auditory material, videos clips), and statements from exemplary people. Prior to implementation, all trainings were adapted to the agricultural setting and target group with regard to content and graphics. After completing the active training phase, participants have the option to conduct a second guided IBI in consultation with the e-coach. (3) The final *maintenance phase* enables monthly e-coach contact *via* text or telephone for up to 12 months.

#### 2.2.2. Personalized tele-based coaching

Personalized tele-based coaching is provided by the company IVPNetworks (www.ivpnetworks.de). SVLFG call center agents register participants on the IVPNetworks management and documentation platform (IVPnet 2.0), and a case manager at IVPNetworks assigns the participant to a coach. The coaches are psychologists with training in different psychological methods (e.g., systemic therapy, psychodynamic psychology, hypnotherapy, CBT) and supervision by licensed psychotherapists. There are no standardized manuals for the coaching. Coaching methods depend on the coach’s professional and therapeutic background. According to the coaches, the TC can generally be described to entail an “initial phase” (e.g., contact bilding and problem definition), a “working phase” (e.g., problem solving) and a “stabilizing phase” (maintaining effects) (30). The coaching is personalized in terms of number, duration, and frequency of the sessions, depending on the individual participant’s needs. A period of 850 min (or 6 months) is set as the maximum, with the possibility of an extension of another 150 min (or 3 months). Participants can be supported in finding onsite social care services (e.g., agricultural socioeconomic or family counseling) and, alternatively, if indicated, in using onsite coaching.

### 2.3. Recruitment and data collection

Employees from the following fields at the SVLFG were included in the study:

• Staff involved in the referral process who provide advice on and referral to health services including digital and onsite offers, such as field workers, in-house staff, and call center agents. Since the advice on health offers had to be newly introduced (field workers, in-house staff) or the previous advice had to be adapted or extended to include the digital offers (call center agents), these employees experienced changes in their daily work.
• Staff involved in the strategic planning and conduction of implementation activities (implementation team).

Employees who work as both call center agents as well as implementation team members were excluded from the staff surveys for employees involved in the referral process due to mixed roles, in order to avoid any bias in the data. These employees were only able to participate in the surveys for the implementation team.

Employees were recruited from March 2018 to June 2021 for study participation while engaging in one of six kickoff events in the respective rollout area (e.g., for field workers, in-house staff as well as call center agents) at which the digital offerings and the accompanying implementation study were presented. Since the training of the pilot rollout employees has already taken place in 2017/2018, they were invited to the study *via* their supervisors (by e-mail and at internal meetings) in spring 2019. In total, 365 employees involved in the referral process have been invited for study participation and received an informed consent. The study team sent about two reminder e-mails to the respective group of employees to increase the study enrollment and response rate. In total, 86 employees involved in the referral process (86/365, 23.6%; *n* = 75 field workers and in-house staff; *n* = 11 call center agents) and seven implementation team members (7/7, 100%) signed the informed consent.

An overview of employee surveys and response rates from May 2019 until May 2022 per occupational group and rollout area is illustrated in Table 1. The time of the survey varied depending on the employee group and rollout area (consisting of 2–7 federal states of Germany). Data collection occurred at regular 6-month intervals from May 2019 to May 2022. Employees in the pilot area (Bavaria, Schleswig-Holstein) and Rollout Area A (Bremen, Lower Saxony, Saxony-Anhalt) were surveyed from May 2019 to May 2021, and employees from Rollout Area B (Hesse, North Rhine-Westphalia, Rhineland-Palatinate, Saarland) from November 2019 to May 2021. Employees from Rollout Area C (Baden-Württemberg, Berlin, Brandenburg, Mecklenburg-Western Pomerania, Saxony, and Thuringia) received delayed training due to the COVID-19 pandemic and were therefore surveyed only from May 2021 to May 2022. Therefore, the data collection period was extended from 2 years to 3 and a half years. Across all measurement time points and occupational groups, the response rates varied between 79% and 100% with a mean of 91.1% (SD = 18.8). In addition to the quantitative surveys, the implementation team was interviewed as part of a focus group on the current status of service implementation, their perception on the effectiveness of implementation activities, as well as their perceived barriers and facilitating factors. For the aim of this study, only the answers on barriers and facilitating factors were included. In total, seven focus groups were conducted and recorded by the study coordinator (JF) every 6 months during the implementation study. The duration varied between 42 and 105 min (*M* = 81.29, SD = 20.21).

**Table 1:** Overview of employee surveys and response rates from May 2019 until May 2022 per occupational group and rollout area (*N* = 93).

Time	Measurement point	Occupational group	Invited (*N*)	Responded (*N*)	Response rate (%)	Duration (min.)
**May 2019**	T0	Field worker/in-house staff (pilot rollout area)	17	17	100	38.6 (17.5)
	T0	Field worker/in-house staff (rollout area A)	21	19	91	45.1 (18.3)
	T0	Call center agents	11	11	100	28.0 (13.5)
	T0	Implementation team	6	6	100	14.0 (10.1)
**November 2019**	T0	Field worker/in-house staff (rollout area B)	26	25	96	29.0 (11.9)
	T1	Field worker/in-house staff (rollout area A)	20	16	80	50.5 (19.7)
	T1	Field worker/in-house staff (pilot rollout area)	17	16	94	26.7 (13.4)
	T1	Call center agents	11	11	100	41.9 (24.9)
	T1	Implementation team	6	6	100	12.2 (6.3)
**May 2020**	T1	Field worker/in-house staff (rollout area B)	27	22	82	53.7 (36.9)
	T2	Field worker/in-house staff (rollout area A)	17	15	88	26.3 (15.6)
	T2	Field worker/in-house staff (pilot rollout area)	18	18	100	17.9 (7.8)
	T2	Call center agents	11	10	91	24.4 (14.9)
**November 2020**	T2	Field worker/in-house staff (rollout area B)	27	24	89	21.2 (12.7)
	T3	Field worker/in-house staff (rollout area A)	16	14	88	21.8 (11.7)
	T3	Field worker/in-house staff (pilot rollout area)	18	15	83	18.4 (14.8)
	T3	Call center agents	10	10	100	17.2 (9.3)
**May 2021**	T0	Field worker/in-house staff (rollout area C)	17	14	82	31 (13.7)
	T3	Field worker/in-house staff (rollout area B)	24	19	79	35.7 (24.8)
	T4	Field worker/in-house staff (rollout area A)	16	14	88	38.4 (19.6)
	T4	Field worker/in-house staff (pilot rollout area)	17	15	88	31 (22.4)
	T4	Call center agents	10	8	80	18.6 (16.4)
**November 2021**	T4	Field worker/in-house staff (rollout area C)	17	15	88	58.3 (34.7)
	T5	Call center agents	10	10	100	13.9 (3.22)
**May 2022**	T2	Field worker/in-house staff (rollout area C)	17	14	82	43.7 (23.0)
	T2	Implementation team	7	6	87.5	12.7 (6.3)
	T6	Call center agents	10	10	100	26.1 (10)

Employees from rollout area C received delayed training due to the Covid-19 pandemic and were therefore surveyed only from May 2021 to May 2022. The duration of the survey also includes other items/measurement instruments that are not part of this study.

### 2.4. Outcomes

Table 2 summarizes the 30 of 37 (81.1%) selected CFIR constructs across all five CFIR dimensions, the evaluation methods (surveys, focus group, or reporting data) used for the analysis and the source of items. The CFIR construct selection was based on the research team’s decision as to which constructs were relevant to the agricultural setting. Data from all the participants groups were included for most of the constructs. The surveys consisted of open and close questions from validated questionnaires used in implementation research, adapted items from the CFIR guide (48) as well as from other implementation studies based on the CFIR (44, 49, 53, 57–60) (see Table 2). “Readiness for implementation” among the implementation team as well as staff involved in the referral process was measured by using a German version of the Organizational Readiness for Implementing Change (ORIC; *α* = 0.88–0.92; 12 items; scale 1–5) questionnaire (54). “Leadership engagement” was captured from the staff-perspective with the Implementation Leadership Scale (ILS; *α* = 0.93–0.97; 12 items; scale 1–5) (55, 61) with regard to the implementation team as well as supervisors. The perception of the treatment credibility was assessed among staff involved in the referral process by using items from the Credibility Expectancy Questionnaire (CEQ; *α* = 0.84–0.85, 6 items; scale 1–7) (56) adapted to the interventions and the employees’ perspective within the construct “knowledge and beliefs about the intervention”. Single items from the Discriminant Content Validation (DCV; 32 items; scale 1–7) questionnaire based on the Theoretical Domains Framework Questionnaire (52) were used to assess “access to knowledge and information” (DCV domains: knowledge, training), “compatibility” (DCV domain: compatibility with current working activities), (DCV domain: “self-efficacy” (DCV domain: beliefs about capabilities) and “other personal attributes” (DCV domains: skills, optimism, intentions) among staff involved in the referral process. Single items from a German version of the NoMAD questionnaire (51, 62) were used to assess “available resources” (NoMAD item: contextual integration) and “individual stage of change” (NoMAD items: interactional workability, skill set workability).

**Table 2:** Overview CFIR domains and selected constructs (30/37), group of participants, evaluation methods and source of item.

Construct	Participants	Evaluation methods			Source of item
		Survey	Focus group	Reporting data	
**I. INTERVENTION CHARACTERISTICS**					
Intervention source	implementation team (T2)	x			CFIR guide (48)
Evidence Strength and quality	implementation team (T2)	x			CFIR guide (48)
Relative advantage	implementation team (T2), health care workers (T1)	x			CFIR guide (48)
Trialability	implementation team (T2)	x			CFIR guide (48)
Complexity	implementation team (T2)	x			CFIR guide (48), Kegler et al. (49)
Design quality and packaging	implementation team (T2)	x			CFIR guide (48),
Cost	implementation team (T2)	x			Pankratz, Hallfors and Cho (50)
**II. OUTER SETTING**					
Patient needs and resources	implementation team (T2)	x			CFIR guide (48)
External policy and incentives	implementation team (T2)	x			CFIR guide (48)
**III. INNER SETTING**					
Structural characteristics	implementation team (T2)	x			Created
Networks and communications	implementation team (T2)	x			CFIR guide (48)
Implementation climate	implementation team (T2)	x			CFIR guide (48)
Tension for change	implementation team (T2)	x			Created
Compatibility	implementation team (T2), health care workers (T1)	x			Finch et al. (51); Huijg et al. (52)
Relative priority	implementation team (T2)	x			CFIR guide (48)
Goals and feedback	implementation team (T2)	x			Helfrich et al. (53)
Readiness for implementation	implementation team (T1), health care workers (T1)	x			Shea et al. (54)
Leadership engagement	health care workers (T2, T4 or T6)	x			Aarons et al. (55)
Available resources	implementation team (T2), health care workers (T2, T4 or T6)	x			Finch et al. (51); Huijg et al. (52)
Access to knowledge and information	health care workers (May 2021: T0, T3 or T4)	x			Finch et al. (51); Huijg et al. (52)
**IV. CHARACTERISTICS OF INDIVIDUALS**					
Knowledge and beliefs about the intervention	health care workers (T1)	x			Huijg et al. (52); Devilly et al. (56)
Self-efficacy	health care workers (T1)	x			Huijg et al. (52)
Individual stage of change	health care workers (May 2021: T0, T3 or T4)	x			Finch et al. (51)
Other personal attributes	health care workers (T1)	x			Huijg et al. (52)
**V. PROCESS**					
Planning	implementation team (T2)	x			Hadjistavropoulos et al. (44)
Engaging	implementation team (T2)	x	x	x	CFIR guide (48), created
Opinion leaders	implementation team (T2)	x			CFIR guide (48)
Formally appointed internal Implementation leaders	implementation team (T2)	x			CFIR guide (48)
Champions	implementation team (T2)	x			Damschroder et al. (57)
External change agents	implementation team (T2)	x			CFIR guide (48)
Executing	implementation team (T2)	x	x		CFIR guide (48)
Reflecting and evaluating	implementation team (T2)	x	x		Helfrich et al. (53), Jaen et al. (58), CFIR guide (48), Sohng et al. (59)

Health care workers include field worker, in-house staff, and call center agents at the social insurance company. The constructs “adaptability”, “cosmopolitanism”, “culture”, “individual identification with organization”, “learning climate”, “organizational incentives and rewards”, and “peer pressure” were not captured.

Furthermore, we used reporting data on dissemination activities documented by the SVLFG to measure “engaging”. Open questions on barriers and facilitating factors in the implementation (see Supplementary Material) were asked from the second measurement time point (T1) every 6 months in the regular surveys given to staff involved in the referral process as well as to the focus groups with the implementation team whose responses could relate to all five CFIR dimensions.

### 2.5. Analysis

Descriptive analyses were performed to assess outcomes across the CFIR dimensions. The outcomes within the dimension “intervention characteristics” as well as CEQ and DCV questionnaires and single items of the evaluation survey were evaluated separately for the internet- and tele-based interventions. All other outcomes were analyzed jointly. Items with a 6- or 7-level Likert scale (e.g., DCV items, CEQ items 1–3 and 5, items regarding relative advantage) were transformed into a 5-level Likert scale based on linear transformation (63). As in another CFIR study (44), scores of 4 or above were interpreted as positive aspects in the implementation, while scores below 3 were classified as areas for improvement, and scores in between were considered as neutral. However, this does not apply to the cost and complexity items, where higher agreement suggests higher cost or complexity. The analyses were performed per employee group. All observed data were included according to the intention-to-treat principle. Since the response rates (see Table 1) and data quality was relatively high in this implementation study, missing data was not imputed. The analyses were done with R statistic software (64).

Dissemination activities were categorized by two independent raters (JB, IW) according to the Cochrane Effective Practice and Organization of Care (EPOC) taxonomy (65). Each activity could contain multiple strategies. Inconsistent cases were discussed with the third researcher (JF) until all dissemination activities could be consistently assigned.

Answers to open questions on barriers and facilitating factors in the employee surveys were evaluated by two independent raters using a deductive-inductive approach with MAXQDA (66), a software for qualitative analysis. A code system with main categories based on the CFIR (deductive approach) and identified subthemes from the data (inductive approach) was created by a research assistant (JB) to match the data and developed further in feedback loops with two experts in e-mental health and qualitative research (JF, IT). As a text passage could consist of different aspects, it could be coded with several codes. The data were assigned to the code system and frequencies were calculated in relation to the mentions of the individual themes as well as per occupational group [i.e., (a) field workers and in-house staff or (b) call center agents]. The agreement rate between the two independent raters (JB, SM) can be characterized as high (*k* = 0.86) (67).

Based on the audio recordings of the focus groups minutes were taken for each focus group with the implementation team. Answers to open questions regarding barriers and facilitating factors were summarized and analyzed per focus group based on a code system following a deductive-inductive approach. The theoretically based superordinate categories formed the basic framework for the inductively identified topics. Frequencies of themes were analyzed at interview level. The analysis was performed by a research assistant (IW) in an iterative process with several feedback loops with input from clinical researchers (IT, JF).

## 3. Results

### 3.1. Participant characteristics

Most of the 93 study participants were involved in the referral process as field workers and in-house staff (*n* = 75, 80.6%) as well as call center agents (*n* = 11, 11.8%). Seven employees were part of the implementation team at the insurance company (7.5%). On average, the participants were 49.29 years old (SD = 8.6), mostly male (*n* = 63, 67.7%) and worked for more than 10 years at SVLFG (*n* = 61, 65.6%). Most of the field worker and in-house staff had experience with consultations on health offers (*N* = 60, 69.8%), however only 31.5% (*n* = 29) of them had experience with consultations on mental health services. Further characteristics of the study participants are shown in Table 3.

**Table 3:** Sociodemographic data of the employees participating in the implementation study, presented by occupational group and the overall sample (*N* = 93).

Characteristics	Implementation team (*n* = 7)	Field worker and in-house staff (*n* = 75)	Call centre agents (*n* = 11)	Overall sample (*N* = 93)
Age *M* (SD)	41.29 (10.1)	49.88 (8.2)	50.55 (8.7)	49.29 (8.6)
Gender *n* (%)				
Female	4 (57.1)	17 (22.7)	9 (81.8)	30 (32.3)
Male	3 (42.9)	58 (77.3)	2 (18.2)	63 (67.7)
Years worked at the company				
Less than 1 year	0 (0.0)	0 (0.0)	0 (0.0)	0 (0.0)
1–2 years	0 (0.0)	2 (2.7)	0 (0.0)	2 (2.2)
2–3 years	0 (0.0)	0 (0.0)	0 (0.0)	0 (0.0)
3–5 years	0 (0.0)	1 (1.3)	0 (0.0)	1 (1.1)
5–10 years	2 (28.6)	8 (10.7)	1 (9.1)	11 (12.0)
10–20 years	2 (28.6)	25 (33.3)	1 (9.1)	28 (30.1)
20–30 years	2 (28.6)	24 (32.0)	4 (36.4)	30 (32.6)
30–40 years	1 (14.3)	15 (20.0)	5 (45.5)	21 (22.8)
More than 40 years	0 (0.0)	0 (0.0)	0 (0.0)	0 (0.0)
Role at company				
Field worker	--	65 (86.7)	--	--
In-house staff	--	4 (5.3)	--	--
Consultant in a similar field	--	6 (8.0)	--	--
Consultation on health offers in the past (*n* = 78)				
Yes	--	50 (72.3)	7 (77.8)	57 (73.1)
No	--	19 (27.5)	2 (22.2)	21 (26.9)
Consultation on mental health services in the past (*n* = 50)				
Yes	--	29 (58.0)	--	--
No	--	21 (42.0)	--	--

### 3.2. CFIR constructs

**Figure 2:**
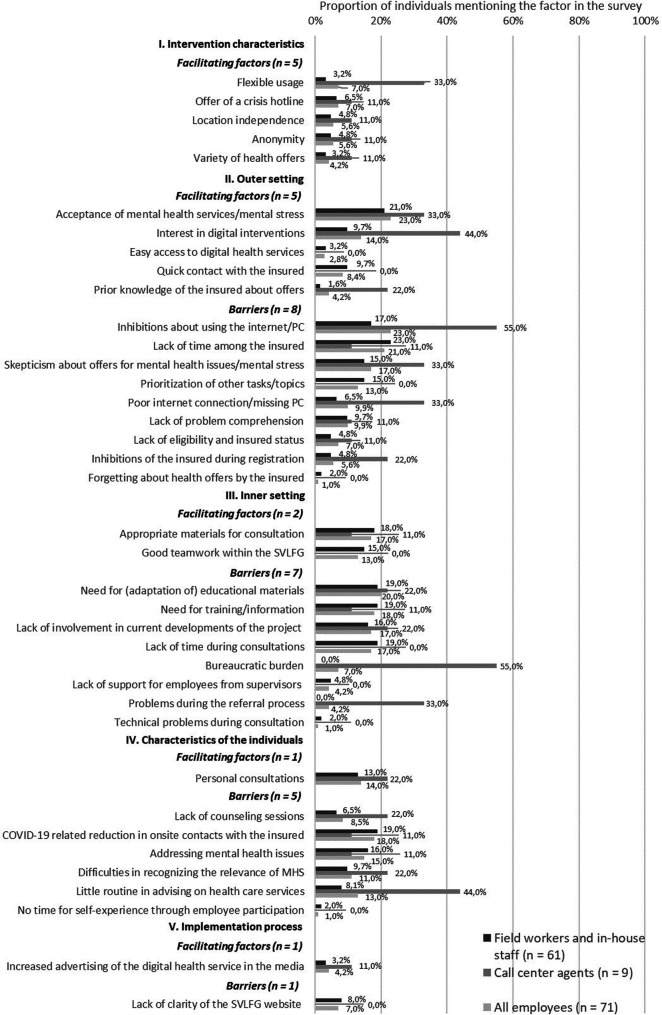
Barriers and facilitating factors from the perspective of staff involved in the referral process (field workers and in-house staff *n* = 62; call center agents *n* = 9), on implementation determinants based on CFIR. MHS, mental health services.

**Figure 3:**
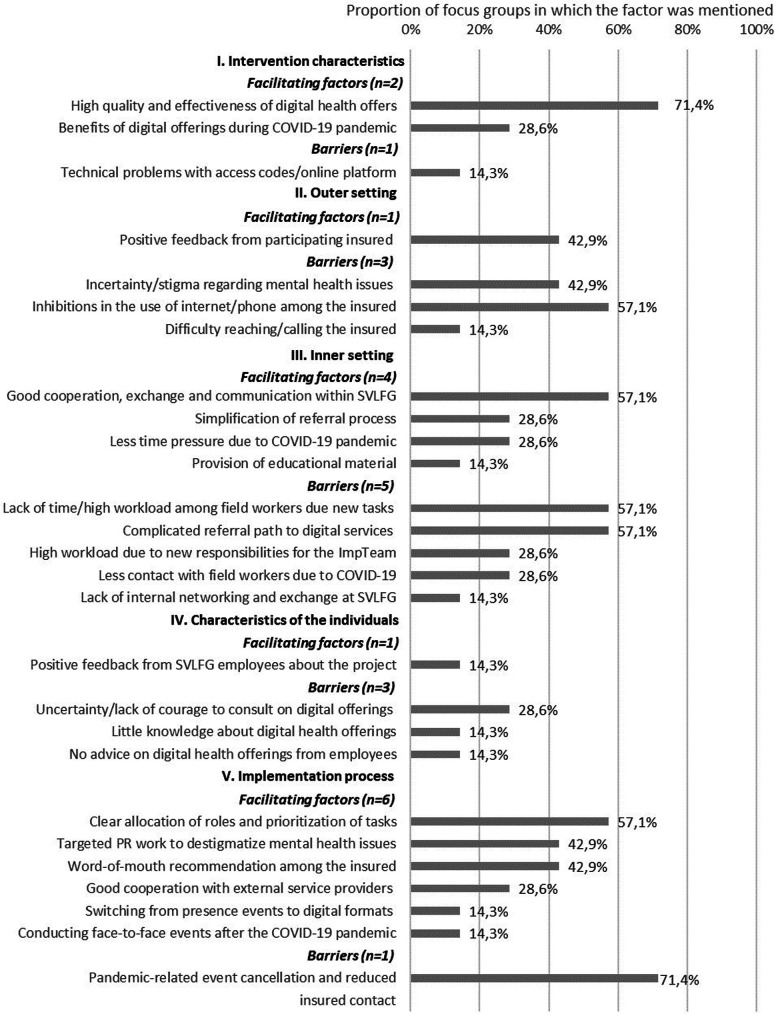
Barriers and facilitating factors from the perspective of the implementation team (*n* = 7) on implementation determinants based on five CFIR dimensions.

In the following, the findings are summarized by the CFIR domain. Each CFIR domain is divided into qualitative and quantitative results. Qualitative findings on barriers (*n* = 20) and facilitating factors (*n* = 10) from the perspective of staff involved in the referral process are illustrated in Figure 2 and as Supplementary Table, including a definition and quotation of the identified theme. Barriers (*n* = 13) and facilitating factors (*n* = 14) from focus groups with the implementation team are shown in Figure 3. Quantitative results on CFIR items among the implementation team are displayed in Table 4, for employees involved in the referral process in Table 5. Descriptive differences between the two interventions as well as between the different types of employees are pointed out.

**Table 4:** Descriptive data of the implementation team on implementation determinants based on CFIR dimensions (*n* = 6).

Items per CFIR construct	Implementation team (*n* = 6) *M* (SD)
**I. INTERVENTION CHARACTERISTICS**	
*Evidence Strength & quality (per intervention)*	
The research evidence on effectiveness supports my belief that the *online health trainings* are effective.	4.67 (0.52)
The research evidence on effectiveness supports my belief that the *personalized tele-based coaching* is effective.	5.00 (0.0)
I assume that influential stakeholders (i.e., key groups of people or stakeholders involved in the implementation) are convinced of the effectiveness of the *online health trainings*.	4.00 (1.10)
I assume that influential stakeholders (i.e., key groups of people or stakeholders involved in the implementation) are convinced of the effectiveness of the *personalized tele-based coaching*.	4.50 (0.84)
*Trialability*	
It was advantageous that the internet- and tele-based health services were implemented in selected pilot regions in Bavaria and Schleswig-Holstein before the nationwide rollout.	4.20 (0.84)
*Complexity*	
It is/was difficult to train employees to implement the internet and tele-supported health services.	3.60 (1.14)
Overall, I believe that implementing internet- and tele-based health services is a complex and challenging process.	3.67 (1.03)
*Design quality & packaging*	
How helpful did you find available tools (e.g., online resources, marketing materials, etc.) to help you implement and use internet- and tele-based health services?	3.11 (1.78)
*Cost*	
The average cost of the *online trainings* per participant is high.	3.00 (1.22)
The average cost of the *personalized tele-based coaching* per participant is high.	3.20 (1.30)
The costs for the implementation of the internet- and tele-based interventions are high.	3.40 (1.14)
*Relative advantage*	
When you consider the advantages and disadvantages of the *online health trainings*, how would you rate the situation overall?	3.67 (0.97)
When you consider the advantages and disadvantages of the *personalized tele-based coaching*, how would you rate the situation overall?	4.73 (0.41)
**II. OUTER SETTING**	
*Patient needs & resources*	
The needs and preferences of the insured were taken into account in the decision to implement internet- and tele-based health services.	4.6 (0.55)
SVLFG employees are aware of the needs and preferences of the insured persons they counsel as part of their official duties.	4.33 (0.82)
*External policy & incentives*	
Regional, state or national policies, as well as legal requirements, have significantly influenced the decision to implement internet- and tele-based health services.	4.25 (1.50)
**III. INNER SETTING**	
*Structural characteristics*	
In your view, to what extent do the SVLFG infrastructure features hinder or facilitate the implementation of internet- and tele-based health services?	2.0 (1.55)
*Networks & communications*	
I perceive the working relationship with colleagues in my department as good.	3.83 (0.98)
I perceive the working relationship with colleagues in other departments as good.	4.00 (0.0)
*Implementation climate*	
Overall readiness for the implementation of internet- and tele-based health services at SVLFG is high.	3.17 (1.17)
*Tension for change*	
There is a high demand for internet- and tele-based health services to counteract mental stress among the insured.	4.50 (0.55)
Internet- and tele-based health care offerings are important to meet the needs of the insured or other organizational goals.	4.67 (0.52)
*Compatibility*	
The *online health trainings* fit my values and norms.	4.33 (0.52)
The *personalized tele-based coaching* fits my values and norms.	4.67 (0.52)
The *online health trainings* fit the values and standards within SVLFG.	4.00 (1.10)
The *personalized tele-based coaching* fits the values and norms within SVLFG.	4.17 (1.17)
The *online health trainings* fit with existing work processes and practices in my environment.	3.83 (0.41)
The *personalized tele-based coaching* fits with existing work processes and practices in my environment.	4.50 (0.55)
*Relative priority*	
The implementation of internet and tele-based health services is a high priority compared to other initiatives at SVLFG that take place throughout the implementation period.	4.40 (0.55)
*Available resources*	
SVLFG has sufficient resources to implement and manage internet- and tele-based health services.	3.67 (1.37)
The implementation team has sufficient staff support needed to implement the internet and tele-based health services.	3.33 (1.37)
*Access to knowledge & information*	
SVLFG employees received adequate training on internet and tele-based health services.	4.0 (1.10)
**IV. CHARACTERISTICS OF INDIVIDUALS**	
No items were recorded for this dimension.	
**V. PROCESS**	
*Planning*	
We have spent sufficient time planning the implementation of internet- and tele-based health services.	3.60 (1.52)
The plan for implementing internet- and tele-based health services is detailed.	3.60 (1.14)
The plan to implement internet- and tele-based health services is/was realistic and feasible.	2.83 (0.98)
Implementation team members have clearly defined roles and responsibilities.	3.17 (1.33)
*Engaging*	
SVLFG has ensured that all employees have been informed about internet and tele-based health services, including those who do not offer counselling on them.	2.67 (1.21)
*Formally appointed internal implementation leaders*	
Does this person [the formally appointed internet implementation leader] have sufficient authority to implement the internet and tele-based health services? (yes/no)	100% (0.0)
*Champions*	
Some SVLFG employees (e.g., field, office, and call center staff) actively support and promote internet- and tele-based health services beyond what is required.	4.0 (1.10)
*External change agents*	
How helpful did you find the support outside SVLFG (e.g., external service providers, associations, and other organizations)?	2.08 (0.74)
*Executing*	
Internet- and tele-based health services were implemented according to the established plan.	3.00 (1.41)
*Reflecting & evaluating*	
There are clear goals within SVLFG or the implementation team for the implementation of internet and tele-supported health services.	3.33 (1.03)
We use data from insured persons to improve care.	3.50 (1.22)
We use data to guide the implementation of internet- and tele-supported health services.	2.83 (1.83)
There is frequent communication within SVLFG about how various change processes are underway as part of the implementation of internet- and tele-supported health services.	3.50 (1.22)

Scores of 4 or above can be interpreted as positive aspects in the implementation process, while scores below 3 were classified as areas for improvement (except for the items regarding to cost and complexity, where higher agreement suggests higher cost or complexity).

**Table 5:** Descriptive data of employees involved in the referral process on implementation determinants based on CFIR dimensions (*n* = 89).

Item	Call center agents *M* (SD)	*N*	Field worker *M* (SD)	*N*	Total *M* (SD)	*N*
**I. INTERVENTION CHARACTERISTICS**						
*Relative advantage*						
When you consider the advantages and disadvantages of the *online health trainings*, how would you rate the situation overall?	3.47 (0.76)	11	3.77 (0.70)	77	3.73 (0.71)	87
When you consider the advantages and disadvantages of the *personalized tele-based coaching*, how would you rate the situation overall?	4.2 (0.62)	11	3.77 (0.65)	77	3.83 (0.66)	87
**II. OUTER SETTING**						
No items were recorded for this dimension.						
**III. INNER SETTING**						
*Compatibility*						
Providing advice on *online health trainings* during company visits or by telephone is compatible with my job as a call center employee or field or office employee.	4.21 (0.93)	11	3.64 (1.07)	67	3.72 (1.06)	78^a^
Providing advice on the *personalized tele-based coaching* during company visits or to provide advice by telephone is compatible with my job as a call center employee or field or office employee.	4.21 (0.93)	11	3.48 (1.08)	67	3.57 (1.06)	78^a^
*Readiness for implementation*						
Organisational Readiness for Implementing Change: Efficacy^b^	3.71 (0.46)	11	3.43 (0.68)	78	3.57 (0.63)	89
Organisational Readiness for Implementing Change: Commitment^b^	3.80 (0.55)	11	3.55 (0.65)	78	3.71 (0.60)	89
Organisational Readiness for Implementing Change (total score)^b^	3.68 (0.45)	11	3.48 (0.64)	78	3.63 (0.58)	89
*Leadership engagement*						
Implementation Leadership (direct supervisor)^b^	4.15 (0.53)	10	2.90 (0.76)	53	3.09 (0.86)	63
Implementation Leadership (implementation team)^b^	3.93 (0.50)	10	3.36 (0.50)	53	3.51 (0.69)	63
*Available resources*						
Sufficient resources are available to implement internet- and tele-based health services.	3.75 (0.71)	10	2.84 (1.05)	61	2.94 (1.05)	71
**IV. CHARACTERISTICS OF INDIVIDUALS**						
*Knowledge & beliefs about the intervention*						
I am aware of the content and goals of the *online health trainings*.	4.39 (0.63)	11	3.62 (0.86)	67	3.73 (0.87)	78^a^
I am aware of the content and goals of *personalized tele-based coaching*.	4.39 (0.70)	11	3.31 (0.89)	67	3.46 (0.94)	78^a^
I know the content and goals of the *online health trainings*.	4.27 (0.70)	11	3.51 (0.84)	67	3.62 (0.86)	78^a^
I know the content and objectives of *personalized tele-based coaching*.	4.27 (0.66)	11	3.20 (0.95)	67	3.36 (1.00)	78^a^
I am familiar with the content and goals of the *online health trainings*.	4.15 (0.85)	11	3.32 (0.89)	67	3.44 (0.93)	78^a^
I am familiar with the content and goals of *personalized tele-based coaching*.	4.27 (0.76)	11	3.06 (1.00)	67	3.23 (1.06)	78^a^
Credibility Expectancy Questionnaire (items 1–3, 5): *online health trainings*^b^	4.48 (1.15)	10	4.44 (0.91)	62	4.44 (0.94)	72
Credibility Expectancy Questionnaire (items 1–3, 5): *personalized tele-based coaching*^b^	4.93 (1.17)	10	4.54 (0.90)	62	4.59 (0.94)	72
Credibility Expectancy Questionnaire (item 4): By the end of the *online health trainings*, how much improvement of mental well-being of the insured do you think will occur?	49.5% (26.5)	10	50.60% (20.3)	62	50.44% (21.1)	72
Credibility Expectancy Questionnaire (item 4): By the end of the *personalized tele-based coaching*, how much improvement of mental well-being of the insured do you think will occur?	60.7% (27.6)	10	55.27% (17.3)	62	56.03% (18.9)	72
Credibility Expectancy Questionnaire (item 6): By the end of the *online health trainings*, how much improvement of mental well-being of the insured do you really feel will occur?	47.4% (26.6)	10	50.08% (21.6)	62	49.71% (22.2)	72
Credibility Expectancy Questionnaire (item 6): By the end of the *personalized tele-based coaching*, how much improvement of mental well-being of the insured do you really feel will occur?	59.9% (28.6)	10	54.97% (19.4)	62	55.65% (20.7)	72
*Self-efficacy*						
I am confident that I can advise insureds about *online health trainings* during site visits or by phone, even if insureds are not motivated.	3.97 (1.05)	11	2.91 (1.12)	67	3.06 (1.16)	78^a^
I am confident in my ability to counsel insureds during facility visits or by telephone for *personalized tele-based coaching*, even if insureds are not motivated.	4.09 (1.09)	11	2.73 (1.02)	67	2.92 (1.13)	78^a^
I am confident that I can advise insureds on *online health trainings* during site visits, even if there is little time.	3.91 (0.91)	11	2.78 (1.14)	67	2.94 (1.17)	78^a^
I am confident in my ability to advise insureds on *personalized tele-based coaching* during facility visits, even when there is little time.	4.15 (0.90)	11	2.72 (1.03)	67	2.92 (1.12)	78^a^
I am confident that I could advise insureds on *online health trainings* during site visits or by phone if I wanted to.	4.88 (0.93)	11	3.40 (1.10)	67	3.51 (1.11)	78^a^
I am confident that I could advise insureds during facility visits or by telephone for *personalized tele-based coaching* if I wanted to.	4.21 (0.83)	11	3.17 (1.04)	67	3.32 (1.07)	78^a^
*Individual stage of change*						
I can easily integrate the internet- and tele-based interventions into my existing work.	3.13 (1.13)	10	3.38 (0.96)	61	3.36 (0.98)	71
Work is assigned to those with skills appropriate to the the internet- and tele-based interventions.	3.50 (0.93)	10	3.97 (0.87)	61	3.91 (0.90)	71
Sufficient training is provided to enable staff to implement the internet- and tele-based interventions.	4.13 (0.35)	10	3.16 (1.01)	61	3.27 (1.01)	71
*Other personal attributes*						
I am skilled at advising insureds on online health trainings during site visits or by telephone.	4.15 (0.79)	11	3.37 (0.97)	77	3.48 (0.98)	78^a^
I am skilled at advising insureds on personalized tele-based coaching during facility visits or by telephone.	4.21 (0.83)	11	3.24 (1.01)	77	3.38 (1.04)	78^a^
In the next 4 weeks, I will in any case advise the insured persons about the online health trainings courses during the company visits or by telephone.	3.79 (1.02)	11	3.39 (1.78)	77	3.44 (1.78)	78^a^
In the next 4 weeks, I will advise the insured in every case during the company visits or by telephone for personalized tele-based coaching.	3.91 (0.80)	11	3.52 (1.63)	77	3.78 (1.70)	78^a^
How strong is your intention to advise insureds about online health trainings during site visits or by telephone?	3.73 (1.17)	11	3.47 (0.97)	77	3.50 (1.00)	78^a^
How strong is your intention to advise insureds during site visits or by telephone on personalized tele-based coaching?	4.03 (0.91)	11	3.03 (1.11)	77	3.17 (1.13)	78^a^
**V. PROCESS**						
No items were recorded for this dimension.						

^a^ Due to an error in the programming of the online survey, not all employees received the item.

^b^ Instead of a single item, several items or a complete measurement were used to assess the respective construct.

#### 3.2.1. Intervention characteristics

##### 3.2.1.1. Qualitative findings

Staff involved in the referral process described five facilitating factors regarding intervention characteristics. Namely, they reported that advantages lie in the “flexible usage” (5/71, 7.0%), the “offer of a crisis hotline” (5/71, 7.0%), “location independence” (4/71, 5.6%), “anonymity” (4/71, 5.6%) of the digital interventions, and the “variety of health offers” (3/71, 5.6%). Then, the implementation team described the “high quality and effectiveness of digital health offers” (5/7, 71.4%) and “benefits of digital offerings during the COVID-19 pandemic” (2/7, 28.6%) as facilitating factors and “technical problems with access codes/online-platform” (1/7, 14.3%) as a barrier to implementation.

##### 3.2.1.2. Quantitative findings

Overall, the average ratings among all employees can be described as high, indicating that intervention characteristics facilitate the implementation. The ratings among the implementation team revealed strong research evidence and the benefit of a pilot implementation phase with both mean ratings above 4.20. Costs for the implementation were rated as rather high (*M* = 3.40), and the implementation was described as a rather complex process across all items (*M* ≥ 3.60). Among the implementation team and call center agents, the relative advantage of the tele-based coaching (4.20 ≤ *M* ≥ 4.73) was rated as high and that of the IBI program as neutral (3.47 ≤ *M* ≥ 3.67) in these occupational groups, while field workers rated both digital interventions on an equal level (*M* = 3.77).

#### 3.2.2. Outer setting

##### 3.2.2.1. Qualitative findings

Five facilitating factors and eight barriers emerged with regard to the outer setting from the perspective of staff involved in the referral process. Employees perceived an “acceptance of mental health services” (16/71, 23.0%) as well as an “interest in digital interventions” (10/71, 14.0%) among the insured. They saw the “easy access to digital health services” (2/71, 2.8%), “quick contact with the insured” (6/71, 8.4%) and “prior knowledge about health offers among the insured” (3/71, 4.2%) as helpful. At the same time, they described “inhibitions about using the internet/PC” (16/71, 23.0%), a “lack of time among the insured” (21/71, 21.0%), “skepticism about offers for mental strain” (12/71, 17.0%), and “prioritization of other tasks/topics” (9/71, 13.0%) among the insured. Further barriers were a “poor internet connection/missing PC” (7/71, 9.9%), a “lack of problem comprehension” (7/71, 9.9%), a “lack of access authorization/insured status” (5/71, 7.0%), “inhibitions during registration” (4/71, 5.6%), and “forgetting about health offers” (1/71, 1.0%) among the insured. In focus groups with the implementation team, “positive feedback from participating insured persons” (3/7, 42.9%) was described as a facilitating factor for the implementation. At the same time, three barriers were identified: “incertainty and stigma regarding mental health issues among the insured” (3/7, 42.9%), “inhibitions about using the internet/phone” (4/7, 57.1%), and “difficulty reaching/calling the insured” (1/7, 14.3%).

##### 3.2.2.2. Quantitative findings

The mean ratings of items assessing the outer setting from the perspective of the implementation team were above 4.25, indicating that this domain facilitates service implementation. The ratings of the implementation team indicate that the needs and preferences of the insured were taken into account in the decision to implement internet- and tele-based health services and that employees involved in the referral process were aware of these needs and preferences.

#### 3.2.3. Inner setting

##### 3.2.3.1. Qualitative findings

Two facilitating factors and seven barriers regarding the inner setting were identified among employees involved in the referral process. The participants emphasized the availability of “appropriate materials for consultation” (12/71, 17.0%), and the “good teamwork within the SVLFG” (9/71, 13.0%). However, a “need for (adaptation of) educational materials” (14/71, 20.0%), “need for training/information” (13/71, 18.0%), a “lack of support from supervisors” (3/71, 4.2%), and “lack of involvement in current developments of the project” (12/71, 17.0%) were reported. Other identified barriers were “lack of time during consultations” (12/71, 17.0%), “bureaucratic burden” (5/71, 7.0%), “problems during the referral process” (3/71, 4.2%), and “technical problems during consultation” (1/71, 1.0%). Four facilitating and five hindering factors were reported in the focus groups with the implementation team. They described “good cooperation, exchange and communication within the SVLFG” (5/7, 57.1%), “simplification of referral process” (2/7, 28.6%), “less time pressure due to the COVID-19 pandemic” (2/7, 28.6%) as many events had to be cancelled due to the pandemic, and the “provision of education material” (1/7, 14.3%) as helpful. Barriers pointed out by the implementation team were a “lack of time and high workload among field workers due to news tasks” (5/7, 57.1%), “complicated referral path to digital services” (5/7, 57.1%), “high workload due to new responsibilities for the implementation team” (2/7, 28.6%), “less contact with field workers due to COVID-19 pandemic” (2/7, 28.6%), and “lack of internal networking and exchange at SVLFG” (1/7, 14.3%).

##### 3.2.3.2. Quantitative findings

In the results on the implementation team perspective, many factors were rated with neutral to high agreement (3.80 ≤ *M* ≥ 4.80), especially networks and communications, tension for change, compatibility with values and existing working processes, relative priority of the digital interventions, and access to knowledge and information. Structural characteristics were rated as hindering (*M* = 2.0). Among staff involved in the referral process, readiness for implementation was evaluted as neutral with means of 3.48 for field workers and 3.68 for call center agents. Leadership engagement with regard to the direct supervisor (*M* = 4.15) and the implementation team supervisor (*M* = 3.90) was perceived as neutral to high by call center agents. Among field workers, leadership engagement from the direct supervisor (*M* = 2.90) and the implementation team (*M* = 3.36) were rated as “in need of improvement” to “neutral”. The same also applies to the ratings of availability of sufficient resources by field workers (2.84 ≤ *M* ≥ 3.75).

#### 3.2.4. Characteristics of the individuals

##### 3.2.4.1. Qualitative findings

The analysis revealed one facilitating factor and five barriers regarding characteristics of the individuals among employees involved in the referral process. The employees described “personal consultations” (10/71, 14.0%) as beneficial. At the same time, they pointed to barriers such as the “COVID-19 related reduction in onsite contacts with insured persons” (13/71, 18.0%), “lack of consultations” (6/71, 8.5%), “addressing mental health issues” (11/71, 15.0%), “little routine in advising on health care services” (9/71, 13.0%), “difficulties in recognizing the relevance of mental health issues” (8/71, 11.0%), and “no time for self-experience through employee participation” (1/71, 1.0%). The implementation team mentioned one facilitator and three barriers in relation to this dimension. Specifically, they described the benefit of “positive feedback from SVLFG employees about the project” (1/7, 14.3%) and the hindrances of “uncertainty and lack of courage to consult on digital health offerings among employees” (2/7, 28.6%), “little knowledge about digital health offerings” (1/7, 14.3%), and “no advice on digital health offerings from employees” (1/7, 14.3%).

##### 3.2.4.2. Quantitative findings

Reportings on “knowledge and beliefs about the intervention”, “other personal attributes”, and “individual stage of change” were neutral to high among employees involved in the referral process (3.03 ≤ *M* ≥ 4.93). While ratings on “self-efficacy” revealed neutral to high agreement rates among call center agents (3.91 ≤ *M* ≥ 4.88), most of the answers (4/6) were below 3.0 (2.72 ≤ *M* ≥ 2.91) among field workers, indicating room for improvement. Overall, the credibility of the interventions was rated as high (*M* > 4.44) by employees involved in the referral process. The employees indicated that they expected the interventions to improve symptoms by at least 49.5%.

#### 3.2.5. Implementation process

##### 3.2.5.1. Qualitative findings

The analysis revealed the facilitating factor “increased advertising of the digital health service in the media” (3/71, 4.2%) and a barrier regarding “confusing website of the SVLFG” (5/71, 7.0%) among staff involved in the referral process. In addition, the implementation team described six facilitating factors and one hindering: “clear allocation of roles and prioritization of tasks” (4/7, 57.1%), “targeted public relations work to destigmatize mental health issues” (3/7, 42.9%), “word-of-mouth recommendation among the insured” (3/7, 42.9%), “good cooperation with external service providers” (2/7, 28.6%), “switching from onsite events to digital formats” (1/7, 14.3%), and “conducting face-to-face events after the COVID-19 pandemic” (1/7, 14.3%) were perceived as facilitating factors, while the “pandemic-related cancellation of events and reduced insured contact” (5/7, 71.4%) was a barrier.

##### 3.2.5.2. Quantitative findings

Concerning the implementation process, the average values resulted in a wide range between 2.08 and 4.0 across all constructs. Agreement among the implementation team was highest for “champions” (e.g., individuals who dedicate themselves to supporting the implementation; *M* = 4.0) and lowest for “external change agents” (e.g., individuals outside the SVLFG; *M* = 2.08), and “engaging” (*M* = 2.67) indicating room for improvement.

##### 3.2.5.3. Reporting data

**Figure 4:**
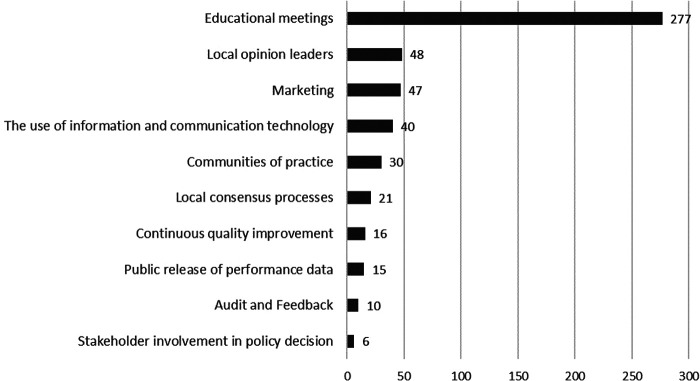
Dissemination activities from April 2019 to September 2022 (*n* = 510), categorized according to the EPOC taxonomy (65). An activity can be assigned to several categories.

As the data showed (see Figure 4), most of the 510 dissemination activities conducted within 3.5 years were classified as strategies in the field of educational meetings (*n* = 277 activities), followed by local opinion leaders (*n* = 48 activities), and marketing (*n* = 47 activities).

## 4. Discussion

Despite the availability of evidence-based digital interventions for the prevention of depression, few interventions find their way into practice. To our knowledge, this is the first study examining the nationwide implementation of internet- and tele-based interventions to prevent depression in a high-risk population (e.g., farmers, forest owners, and gardeners) based on the CFIR (43). The research focus is on facilitating factors and barriers in the implementation of digital interventions from the perspective of health care workers of a social insurance, which helps to elucidate the implementation and to derive recommendations for future adoption and uptake.

### 4.1. Summary

Intervention characteristics seem to have the highest positive impact on the implementation of digital interventions from the perspective of both the implementation team and referral staff. The relative advantage of digital interventions, quality of the interventions, and strength of evidence appeared to be facilitating factors in the implementation of the internet- and tele-based interventions in the agricultural setting.

In comparison, the outer setting, inner setting, individual characteristics, and implementation process were less consistently regarded by all staff groups as having facilitated the implementation of digital prevention services. Most of the qualitatively revealed barriers were related to the outer setting, in particular to “patient needs and resources”. The employees reported to have perceived from the insured, among others, inhibitions about using digital interventions and skepticism about offers for mental strain. At the same time, facilitating factors reflecting the acceptance of mental health services and the general interest of the insured were noted by staff involved in the referral process. Likewise, as indicated by the quantitative ratings for “patient needs and resources” as well as “external policy and incentives”, predominantly beneficial aspects for implementation could be achieved (e.g., consideration of needs and preferences of the insured during implementation; facilitating influence of regional, state or national policies, as well as legal requirements on the implementation).

With regard to the inner setting, many barriers referred to “available resources”, and “access to knowledge and information”, including a need for training and information on the interventions and further educational materials. Although several educational meetings were reported as dissemination activities and kickoff events and training sessions were conducted, there seems to be a need for further information and ongoing training for employees. However, many quantitatively assessed constructs of the inner setting (“tension for change”, “compatibility”, “relative priority”) also served to facilitate the service implementation from the perspective of health care workers.

Further barriers to implementation were identified in relation to the characteristics of individuals. Employees involved in the referral process mentioned having little routine in advising on health care services as well as difficulties in recognizing and addressing mental health issues. The results showed a difference in the agreement rates of the occupational groups. While call center agents evaluated most constructs with high agreements (*M* > 4.0), agreement rates in the group of field workers can be characterized as neutral for “knowledge and beliefs about the intervention” and “other personal attributes”, and as rather hindering for “self-efficacy”.

With regard to the implementation process, facilitating factors were identified in the focus groups with the implementation team, including a clear allocation of tasks and targeted PR activities to destigmatize mental health issues. In the quantitative ratings, facilitating factors related to active implementation support by champions and formally appointed internal implementation leaders and barriers regarding engaging and informing of employees about digital health offerings could be determined by the implementation team. 510 dissemination activities have been carried out so far, most of them in the field of educational meetings, local opinion leaders, and marketing, while others such as strategies like audit and feedback have hardly been used so far.

As there might be differences between the IBI program and TC, some constructs were recorded separately for each intervention. The results for both interventions are generally comparable with neutral to high agreement rates, with slightly higher agreement ratings in favor of the personalized tele-based coaching with regard to “evidence strength and quality”, “relative advantage” and “knowledge and beliefs” about the intervention, particularly evident in the perception of the implementation team and call center agents. However, the current power of the study as well as the unequal number in the groups did not allow for a significance test. As the results of the accompanying randomized controlled trials show (28–30, 34), both interventions effectively reduce depressive symptoms posttreatment.

When interpreting the results, it is important to note that, the COVID-19 pandemic occurred as an unforeseen and uncontrollable event, and the effects are reflected in several constructs. On one hand, kickoff events, exhibition stands, and educational meetings had to be cancelled due to the pandemic, which led to reduced onsite contact with the insured as well as less contact between field workers and the implementation team. On the other hand, digital interventions proved beneficial and important, and these continued to be offered during the pandemic, compared to onsite services. The implementation team indicated that the cancellation of events also eased time pressure, which in turn had a beneficial effect on the planning of new implementation activities. Furthermore, implementation activities could be adapted and planned onsite events were transferred in a digital format. However, the conduction of onsite events from 2022 was also experienced as beneficial by the implementation team.

In the study, different professional groups were interviewed and surveyed. It is particularly evident that there is often an overlap in experiences between professional groups, e.g., both the implementation team and the staff involved in the referral process reported many barriers in relation to the outer and inner setting. In many areas, the results between the professional groups complement each other well (e.g., referral staff who are in daily contact with the insured mentioned even more facilitating factors and barriers regarding “patient needs and resources”). Since the working areas of the employees and therefore the focus, experiences, and tasks in the implementation are different, this contributes to a comprehensive analysis of the implementation process.

### 4.2. Comparison to literature

Although CFIR is one of the most frequently used frameworks to examine implementation in diverse health settings (68), literature in the area of digital interventions to prevent depression is scarce. Hadjistavropoulos et al. (44) conducted a process evaluation based on the CFIR to understand facilitating factors and barriers impacting the implementation of iCBT within community mental health clinics. In line with their study, intervention characteristics were perceived as mostly facilitating the implementation. Similar to our study, the inner setting was identified as the most significant barrier to implementation due to limited resources for internet-based Cognitive Behavioral Therapy (iCBT) (44). In addition, a greater priority given to face-to-face psychotherapy was reported as a barrier to implementation (44), which was not found in our study. Another reported barrier in the implementation of iCBT was the need for even greater engagement of stakeholders in iCBT (44), which also applies for the prevention setting in this study.

Two studies about the implementation of IBIs for depression prevention in adolescents (37) or for depression treatment in adults (69) showed that primary care physicians or nurses perceive the preventive approach of internet interventions as valuable (37). The pilot study (37) reported that it is challenging to train the individuals to achieve a sufficient level of counseling competence, whereas the qualitative study (69) reported a lack of habit and routine and little knowledge about IBIs that could have hindered the referral process and led to low referral rates in primary care physicians. This is consistent with some of the barriers identified in our study related to staff involved in the referral process (e.g., need for training, difficulties in addressing mental health issues, little routine in advising on health care services).

In line with systematic reviews examining factors influencing the implementation of digital interventions (70, 71), our findings indicate that the implementation of digital interventions for depression prevention are influenced by diverse constructs instead of a single construct explaining implementation. Access to knowledge and information seems to be important for the implementation of e-health interventions (70), which was identified as a hindering factor in our study. While access to ongoing system support had a positive impact on implementation, Ross et al. (70) identified it as a barrier to implementation when it was lacking, which is consistent with identified aspects related to training and educational materials in this study.

Most of the identified hindering themes in our study are related to “patient needs and resources”. These barriers are in accordance with previous studies indicating that help-seeking behavior is lower and stigmatization of mental disorders is higher among farmers compared to other occupational groups (25). Furthermore, many of our identified barriers and facilitating factors are in line with findings of qualitative results of another substudy of ImplementIT (31) from the perspective of users (farmers who participated in digital services to prevent depression), including “location independence” and “anonymity” as positive drivers and a “lack of time” and “inhibitions about using the internet” as negative drivers for the acceptance of and satisfaction with the IMI program (17, 32, 33).

### 4.3. Implications

Because of the multifaceted aspects considered in the CFIR, it was a useful framework to explore and map the various contextual factors in the implementation of digital interventions. With the help of the CFIR, adaptations of the digital interventions as well as recommendations for the further implementation and scale-up can be derived. However, we have found an overlap between constructs of the inner setting and individual characteristics as individuals could also be considered part of the inner setting.

Additionally, previous implementation studies on digital interventions for depression identified a lack of methodological rigor and a need for validated questionnaires in the implementation of e-mental health interventions (72). Since no questionnaire exists in which all CFIR constructs are captured, this study provides considerations and initial starting points for data collection and analysis within a mixed-methods approach for other researchers who plan to evaluate the implementation of digital interventions.

Several measures for the further implementation of digital offerings among farmers can be derived from the results. Special attention should be given to addressing barriers regarding “patient needs and resources”. Counteracting the stigmatization of mental stress is a constant and ongoing process, which was also described as beneficial by the implementation team. When implementing technology in the area of prevention, it is often necessary to create an awareness that targeted interventions can increase well-being and significantly reduce the subsequent occurrence of depression. This could be strengthened through targeted press and PR activities, which also refer to the reported positive experiences of participated farmers, and the involvement of local stakeholders. At the level of the inner setting, it could be crucial to conduct (follow-up) trainings for employees in order to provide information about the interventions and current developments and to strengthen the exchange of experiences among each other. In addition to training, other ways of reaching employees at regular intervals should be considered to enhance access to knowledge and information (e.g., newsletter, regular online or onsite meetings). An extension of educational materials (e.g., videos) tailored to the needs of the respective occupational group could be useful to support the consultations. A further strategy according to the CFIR-ERIC Matching Tool (73), which matches barriers to implementation strategies, might be to “involve executive boards” (e.g., supervisors) in the implementation effort to enhance leadership engagement. In order to address barriers with regard to characteristics of the individuals, the trainings should also pay attention to how mental health topics could be addressed or which health offer is useful with the respective insured. Additionally, implementation strategies such as to “conduct ongoing training” for both digital interventions as well as to “make training dynamic” (e.g., by varying the methods of information delivery and making the training interactive to accommodate different learning styles) might strengthen “self-efficacy” as reported in the CFIR-ERIC Matching Tool (73). To enhance “reflecting and evaluating” implementation strategies such as to “develop and implement tools for qualtiy monitoring” as well as to “develop and organize quality monitoring systems” seem to be suitable (73).

For the field of depression prevention, the findings indicate that there are synergies when IBIs and tele-based coachings are implemented as technology-based interventions to complement on-site services. We could identify and address different and common barriers and facilitating factors, while also addressing different preferences and conditions in implementation. Offering different services (individual, group, on-site, tele-based, internet-based) centrally managed by a call center that coordinates inventions from different service providers allows the end-user a lower access threshold with maximum consideration of their needs and could solve the scaling problems observed in research (e.g., low uptakes) (74), where so far usually only one intervention has been implemented and considered. In addition, the findings indicate that it is critical to include, besides end-users, key gatekeepers such as health care workers and their experiences in providing consultations on digital health services as well as implementers at the social insurance company. Further stakeholders in the health care system (e.g., employees/psychologists of the external service providers, political representatives, decision makers of the social insurance) should also be considered, which is partly done in another study by ImplementIT (31) and will be published elsewhere.

### 4.4. Limitations

Our results should be considered in light of the following limitations. First, a smaller sample size among employees than expected could be reached for participation. While 365 employees involved in the consultation process were trained, only 93 of them chose to participate in the study. Furthermore, selection bias may have occurred in that the participants were likely to be more open and motivated to the implementation of digital interventions (selection bias), and positive responses to satisfy the study team (response bias) may occurred as well, which could have resulted in a more positive assessment of service implementation than would have been the case with a representative sample of employees. Second, as no questionnaire exists that covers all CFIR constructs, a mixture of different questionnaires/items with various scales has been used in the study. Therefore, some of the scales had to be transformed during the evaluation in order to be able to present the results uniformly. Third, barriers and facilitating factors were captured generally and not formulated specifically with respect to each CFIR construct, which would have resulted in a larger output for all CFIR dimensions. However, this can also lead to participants responding more openly and less constrained by predetermined deductive-formulated questions. This can be an advantage because more facets outside fixed constructs are raised. Forth, it is unclear what impact the different assessment time points in the respective rollout areas with different characteristics (e.g., regional conditions, team structures, stage of implementation) might have. Fifth, the qualitative results refer to individual responses from employees and cannot be generalized due to the explorative approach.

### 4.5. Conclusion

Our findings contribute to an understanding of the facilitating and hindering factors in the implementation of digital interventions for depression prevention among farmers from the perspective of health care workers and are part of a complex implementation study design (ImplementIT) (31). The study presents that the use of various mixed-methods for data collection including surveys, open-ended questions, focus groups, and reporting data contribute a comprehensive understanding of the implementation process. While both offerings (guided IBIs, personalized tele-based coaching) are widely accepted by health care workers, the results also point to some barriers and contribute to deriving recommendations for further implementation. For example, to improve access to knowledge and information, as well as beliefs about digital prevention services, implementation strategies focusing on educational meetings for employees and distribution of educational materials tailored to the occupational group within the SVLFG might be helpful.

## Data Availability

The raw data supporting the conclusions of this article will be made available by the authors, without undue reservation.

## References

[1] Page H Ayuso-Mateos J Dowrick C Vazquez-Barquero J. Depressive disorders in Europe: morbidity figures from the ODIN study. Br J Psychiatry. (2001) 179(4):308–16. 10.1192/bjp.179.4.308 11581110

[2] World Health Organization. Depression and other common mental disorders: Global health estimates. Geneva: World Health Organization (2017).

[3] Smith M. Stigma. Adv Psychiatr. (2002) 8:317–23. 10.1192/apt.8.5.317

[4] Smith K. Mental health: a world of depression. Nature. (2014) 515(7526):180–1. 10.1038/515180a 25391942

[5] Cuijpers P. Indirect prevention and treatment of depression: an emerging paradigm? Clin Psychol Eur. (2021) 3(4):1–9. 10.32872/cpe.6847 PMC9667226 36398290

[6] Andrews G Issakidis C Sanderson K Corry J Lapsley H. Utilising survey data to inform public policy: comparison of the cost-effectiveness of treatment of ten mental disorders record status study population. Br J Psychiatry. (2019) 184(6):526–33. 10.1192/bjp.184.6.526 15172947

[7] Muñoz RF Cuijpers P Smit F Barrera AZ Leykin Y. Prevention of major depression. Annu Rev Clin Psychol. (2010) 6(1):181–212. 10.1146/annurev-clinpsy-033109-132040 20192789

[8] Martínez-Pérez B de la Torre-Díez I López-Coronado M. Mobile health applications for the most prevalent conditions by the world health organization: review and analysis. J Med Internet Res. (2013) 15(6):e120. 10.2196/jmir.2600 23770578 PMC3713954

[9] Karyotaki E Efthimiou O Miguel C Bermpohl FMG Furukawa TA Cuijpers P Internet-based cognitive behavioral therapy for depression: a systematic review and individual patient data network meta-analysis. JAMA Psychiatry. (2021) 78(4):361–71. 10.1001/jamapsychiatry.2020.4364 33471111 PMC8027916

[10] Lattie EG Adkins EC Winquist N Stiles-Shields C Wafford QE Graham AK. Digital mental health interventions for depression, anxiety, and enhancement of psychological well-being among college students: systematic review. J Med Internet Res. (2019) 21(7):e12869. 10.2196/12869 31333198 PMC6681642

[11] Hussain-Shamsy N Shah A Vigod SN Zaheer J Seto E. Mobile health for perinatal depression and anxiety: scoping review. J Med Internet Res. (2020) 22(4):e17011. 10.2196/17011 32281939 PMC7186872

[12] Etzelmueller A Vis C Karyotaki E Baumeister H Titov N Berking M Effects of internet-based cognitive behavioral therapy in routine care for adults in treatment for depression and anxiety: systematic review and meta-analysis. J Med Internet Res. (2020) 22(8):e18100. 10.2196/18100 32865497 PMC7490682

[13] Ebert DD van Daele T Nordgreen T Karekla M Compare A Zarbo C Internet- and mobile-based psychological interventions: applications, efficacy, and potential for improving mental health. Eur Psychol. (2018) 23(2):167–87. 10.1027/1016-9040/a000318

[14] Andrade LH Alonso J Mneimneh Z Wells JE Al-Hamzawi A Borges G Barriers to mental health treatment: results from the WHO world mental health surveys. Psychol Med. (2014) 44(6):1303–17. 10.1017/S0033291713001943 23931656 PMC4100460

[15] Josephine K Josefine L Philipp D David E Harald B. Internet- and mobile-based depression interventions for people with diagnosed depression: a systematic review and meta-analysis. J Affect Disord. (2017) 223:28–40. 10.1016/j.jad.2017.07.021 28715726

[16] Reins JA Buntrock C Zimmermann J Grund S Harrer M Lehr D Efficacy and moderators of internet-based interventions in adults with subthreshold depression: an individual participant data meta-analysis of randomized controlled trials. Psychother Psychosom. (2021) 90(2):94–106. 10.1159/000507819 32544912

[17] Thielecke J Buntrock C Freund J Braun L Ebert DD Harald B How to promote usage of telehealth interventions for farmers’ mental health? A qualitative study on supporting and hindering aspects for acceptance and satisfaction with a personalized telephone coaching. 10.1016/j.invent.2023.100671 PMC10523267 37772161

[18] Lin T Heckman TG Anderson T. The efficacy of synchronous teletherapy versus in-person therapy: a meta-analysis of randomized clinical trials. Clin Psychol Sci Pract. (2022) 29(2):167. 10.1037/cps0000056

[19] Sue S Keith H Joan F Aslög M. Stress in farmers: a survey of farmers in England and Wales. Occup Environ Med. (1998) 55(11):729–34. 10.1136/oem.55.11.729 9924448 PMC1757527

[20] Sanne B Mykletun A Moen BE Dahl AA Tell GS. Farmers are at risk for anxiety and depression: the hordaland health study. Occup Med. (2004) 54(2):92–100. 10.1093/occmed/kqh007 15020727

[21] Onwuameze OE Paradiso S Peek-Asa C Donham KJ Rautiainen RH. Modifiable risk factors for depressed mood among farmers. Ann Clin Psychiatry. (2013) 25(2):83–90. 23638438

[22] Roy P Tremblay G Oliffe JL Jbilou J Robertson S. Male farmers with mental health disorders: a scoping review. Aust J Rural Health. (2013) 21(1):3–7. 10.1111/ajr.12008 23384130

[23] Logstein B. Predictors of mental complaints among Norwegian male farmers. Occup Med. (2016) 66(4):332–7. 10.1093/occmed/kqw019 26944593

[24] Sanne B Mykletun A Dahl AA Moen BE Tell GS. Occupational differences in levels of anxiety and depression: the hordaland health study. J Occup Environ Med. (2003) 45(6):628–38. 10.1097/01.jom.0000069239.06498.2f 12802216

[25] Judd F Jackson H Fraser C Murray G Robins G Komiti A. Understanding suicide in Australian farmers. Soc Psychiatry Psychiatr Epidemiol. (2006) 41(1):1–10. 10.1007/s00127-005-0007-1 16341827

[26] Torske MO Bjørngaard JH Hilt B Glasscock D Krokstad S. Farmers’ mental health: a longitudinal sibling comparison - the HUNT study, Norway. Scand J Work Environ Heal. (2016) 42(6):547–56. 10.5271/sjweh.3595 27636024

[27] Schang L Schüttig W Sundmacher L. Unterversorgung im ländlichen raum–wahrnehmung der versicherten und ihre präferenzen für innovative versorgungsmodelle. In: Böcken J Braun B Meierjürgen R, editors. Gesundheitsmonitor 2016: bürgerorientierung im gesundheitswesen; kooperationsprojekt der bertelsmann stiftung und der BARMER GEK. Gütersloh: Verlag Bertelsmann Stiftung (2016). p. 58–85.

[28] Braun L Titzler I Terhorst Y Freund J Thielecke J Ebert DD Are guided internet-based interventions for the indicated prevention of depression in green professions effective in the long run? Longitudinal analysis of the 6- and 12-month follow-up of a pragmatic randomized controlled trial (PROD-A). Internet Interv. (2021) 26:100455. 10.1016/j.invent.2021.100455 34900605 PMC8640872

[29] Braun L Titzler I Terhorst Y Freund J Thielecke J Ebert D Effectiveness of guided internet-based interventions in the indicated prevention of depression in green professions (PROD-A): results of a pragmatic randomized controlled trial. J Affect Disord. (2021) 278:658–71. 10.1016/j.jad.2020.09.066 33096333

[30] Thielecke J Buntrock C Titzler I Braun L Freund J Berking M Telephone coaching for the prevention of depression in depression in farmers: results from a pragmatic from a pragmatic randomized controlled trial. J Telemed Telecare. (2022) 0(0):1357633X2211060. 10.1177/1357633X221106027 35695234

[31] Freund J Titzler I Thielecke J Braun L Baumeister H Berking M Implementing internet- and tele-based interventions to prevent mental health disorders in farmers, foresters and gardeners (ImplementIT): study protocol for the multi-level evaluation of a nationwide project. BMC Psychiatry. (2020) 20(1):424. 10.1186/s12888-020-02800-z 32854660 PMC7450981

[32] Braun L Freund J Thielecke J Baumeister H Ebert DD Titzler I. Barriers and facilitators to engaging and adhering to guided internet-based interventions for depression prevention and reduction of pain-related disability in green professions: a mixed methods study. JMIR Ment Heal. (2022) 9(11):e39122. 10.2196/39122 PMC9685507 36350684

[33] Freund J Buntrock C Braun L Thielecke J Baumeister H Berking M Digital prevention of depression for farmers? A qualitative study on participants’ experiences regarding determinants of acceptance and satisfaction with a tailored guided internet intervention program. Internet Interv. (2022) 29:100566. 10.1016/j.invent.2022.100566 36039069 PMC9418375

[34] Braun L Terhorst Y Titzler I Freund J Thielecke J Ebert DD Lessons learned from an attempted pragmatic randomized con-trolled trial for improvement of chronic pain-associated disa-bility in green professions: long-term effectiveness of a guided online-based acceptance and commitment therapy (PACT-A). Int J Environ Res Public Health. (2022) 19(13858):1–24. 10.3390/ijerph192113858 PMC9655679 36360738

[35] Drozd F Vaskinn L Bergsund HB Haga SM Slinning K Bjørkli CA. The implementation of internet interventions for depression: a scoping review. J Med Internet Res. (2016) 18:e5670. 10.2196/jmir.5670 PMC5034149 27608548

[36] Minarik C Moessner M Ozer F Bauer S. Implementierung und dissemination eines internetbasierten programms zur prävention und frühen intervention bei essstörungen. Psychiatr Prax. (2013) 40(06):332–8. 10.1055/s-0033-1349488 24008682

[37] Eisen JC Marko-Holguin M Fogel J Cardenas A Bahn M Bradford N Pilot study of implementation of an internet-based depression prevention intervention (CATCH-IT) for adolescents in 12 US primary care practices: clinical and management/organizational behavioral perspectives. Prim Care Companion CNS Disord. (2013) 15(6):27106. 10.4088/PCC.10m01065 PMC3977759 24800110

[38] Folker AP Mathiasen K Lauridsen SM Stenderup E Dozeman E Folker MP. Implementing internet-delivered cognitive behavior therapy for common mental health disorders: a comparative case study of implementation challenges perceived by therapists and managers in five European internet services. Internet Interv. (2018) 11:60–70. 10.1016/j.invent.2018.02.001 30135761 PMC6084870

[39] Eccles M Grimshaw J Walker A Johnston M Pitts N. Changing the behavior of healthcare professionals: the use of theory in promoting the uptake of research findings. J Clin Epidemiol. (2005) 58(2):107–12. 10.1016/j.jclinepi.2004.09.002 15680740

[40] Grol RPTM Bosch MC Hulscher MEJL Eccles MP Wensing M. Planning and studying improvement in patient care: the use of theoretical perspectives. Milbank Q. (2007) 85(1):93–138. 10.1111/j.1468-0009.2007.00478.x 17319808 PMC2690312

[41] Michie S Johnston M Francis J Hardeman W Eccles M. From theory to intervention: mapping theoretically derived behavioural determinants to behaviour change techniques. Appl Psychol. (2008) 57(4):660–80. 10.1111/j.1464-0597.2008.00341.x

[42] Aarons GA Hurlburt M Horwitz SM. Advancing a conceptual model of evidence-based practice implementation in public service sectors. Adm Policy Ment Health. (2011) 38(1):4–23. 10.1007/s10488-010-0327-7 21197565 PMC3025110

[43] Damschroder LJ Aron DC Keith RE Kirsh SR Alexander JA Lowery JC. Fostering implementation of health services research findings into practice: a consolidated framework for advancing implementation science. Implement Sci. (2009) 4(1):50. 10.1186/1748-5908-4-50 19664226 PMC2736161

[44] Hadjistavropoulos HD Nugent MM Dirkse D Pugh N. Implementation of internet-delivered cognitive behavior therapy within community mental health clinics: a process evaluation using the consolidated framework for implementation research. BMC Psychiatry. (2017) 17(1):1–15. 10.1186/s12888-017-1496-7 28899365 PMC5596488

[45] Pinnock H Barwick M Carpenter CR Eldridge S Grandes G Griffiths CJ Standards for reporting implementation studies (StaRI) statement. Br Med J. (2017) 356(6795):i6795. 10.1136/bmj.i6795 28264797 PMC5421438

[46] Lazarus RS Folkman S. Transactional theory and research on emotions and coping. Eur J Pers. (1987) 1(3):141–69. 10.1002/per.2410010304

[47] Sc H Strosahl K Wilson KG. Acceptance and commitment therapy: an experiential approach to behavior change. New York: Guilford Press (1999).

[48] CFIR guide (2022). Available at: https://cfirguide.org/.

[49] Kegler MC Liang S Weiner BJ Tu SP Friedman DB Glenn BA Measuring constructs of the consolidated framework for implementation research in the context of increasing colorectal cancer screening in federally qualified health center. Health Serv Res. (2018) 53(6):4178–203. 10.1111/1475-6773.13035 30260471 PMC6232399

[50] Pankratz M Hallfors D Cho H. Measuring perceptions of innovation adoption: the diffusion of a federal drug prevention policy. Health Educ Res. (2002) 17(3):315–26. 10.1093/her/17.3.315 12120847

[51] Finch TL Girling M May CR Mair FS Murray E Treweek S Improving the normalization of complex interventions: part 2-validation of the NoMAD instrument for assessing implementation work based on normalization process theory (NPT). BMC Med Res Methodol. (2018) 18(1):135. 10.1186/s12874-018-0591-x 30442094 PMC6238372

[52] Huijg JM Gebhardt WA Crone MR Dusseldorp E Presseau J. Discriminant content validity of a theoretical domains framework questionnaire for use in implementation research. Implement Sci. (2014) 9(11). 10.1186/1748-5908-9-11 PMC3896680 24423394

[53] Helfrich CD Li Y-F Sharp ND Sales AE. Organizational readiness to change assessment (ORCA): development of an instrument based on the promoting action on research in health services (PARIHS) framework. Implement Sci. (2009) 4(1):1–13. 10.1186/1748-5908-4-38 19594942 PMC2716295

[54] Shea CM Jacobs SR Esserman DA Bruce K Weiner BJ. Organizational readiness for implementing change: a psychometric assessment of a new measure. Implement Sci. (2014) 9(1):7. 10.1186/1748-5908-9-7 24410955 PMC3904699

[55] Aarons GA Ehrhart MG Farahnak LR. The implementation leadership scale (ILS): development of a brief measure of unit level implementation leadership. Implement Sci. (2014) 9(1):45. 10.1186/1748-5908-9-45 24731295 PMC4022333

[56] Devilly GJ Borkovec TD. Psychometric properties of the credibility/expectancy questionnaire. J Behav Ther Exp Psychiatry. (2000) 31(2):73–86. 10.1016/S0005-7916(00)00012-4 11132119

[57] Damschroder LJ Hagedorn HJ. A guiding framework and approach for implementation research in substance use disorders treatment. Psychol Addict Behav. (2011) 25(2):194. 10.1037/a0022284 21443291

[58] Jaén CR Crabtree BF Palmer RF Ferrer RL Nutting PA Miller WL Methods for evaluating practice change toward a patient-centered medical home. Ann Fam Med. (2010) 8(Suppl 1):S9–0. 10.1370/afm.1108 20530398 PMC2885721

[59] Sohng HY Kuniyuki A Edelson J Weir RC Song H Tu S-P. Capability for change at community health centers serving Asian pacific islanders: an exploratory study of a cancer screening evidence-based intervention. Asian Pacific J Cancer Prev. (2013) 14(12):7451–7. 10.7314/APJCP.2013.14.12.7451 24460318

[60] `Fernandez M Calo W Kegler M Carvalho M Liang L Weiner B Developing measures to assess constructs from the inner setting of the consolidated framework for implementation research. Implement Sci. (2014) 13:1–13. 10.1186/s13012-018-0736-7 PMC5870186 29587804

[61] Aarons GA Ehrhart MG Torres EM Finn NK Roesch SC. Validation of the implementation leadership scale (ILS) in substance use disorder treatment organizations. J Subst Abuse Treat. (2016) 68:31–5. 10.1016/j.jsat.2016.05.004 27431044 PMC5349507

[62] Elf M Nordmark S Lyhagen J Lindberg I Finch T Åberg AC. The Swedish version of the normalization process theory measure S-NoMAD: translation, adaptation, and pilot testing. Implement Sci. (2018) 13(1):146. 10.1186/s13012-018-0835-5 30509289 PMC6278165

[63] IBM SPSS Statistics. Transforming different Likert scales to a common scale. Available at: https://www.ibm.com/support/pages/transforming-different-likert-scales-common-scale.

[64] R Core Team. R: A language and environment for statistical computing. Vienna, Austria: R Foundation for Statistical Computing (2019). Available at: http://www.r-project.org/.

[65] Grimshaw J Thomas R MacLennan G Fraser C Ramsay CR Vale L Effectiveness and efficiency of guideline dissemination and implementation strategies (2004). 10.3310/hta8060 14960256

[66] VERBI GmbH. MAXQDA 2022. 2022. Available at: https://www.maxqda.de/.

[67] Landis JR Koch GG. The measurement of observer agreement for categorical data. Biometrics. (1977) 33(1):159–74. 10.2307/2529310 843571

[68] Kirk MA Kelley C Yankey N Birken SA Abadie B Damschroder L. A systematic review of the use of the consolidated framework for implementation research. Implement Sci. (2016) 11(1):1–13. 10.1186/s13012-016-0437-z 27189233 PMC4869309

[69] Titzler I Berking M Schlicker S Riper H Ebert DD. Barriers and facilitators for referrals of primary care patients to blended internet-based psychotherapy for depression: mixed methods study of general practitioners’ views. JMIR Ment Heal. (2020) 7(8):e18642. 10.2196/18642 PMC7463410 32673213

[70] Ross J Stevenson F Lau R Murray E. Factors that influence the implementation of e-health: a systematic review of systematic reviews (an update). Implement Sci. (2016) 11(1):1–12. 10.1186/s13012-016-0510-7 27782832 PMC5080780

[71] Vis C Mol M Kleiboer A Bührmann L Finch T Smit J Improving implementation of emental health for mood disorders in routine practice: systematic review of barriers and facilitating factors. J Med Internet Res. (2018) 20:e9769. 10.2196/mental.9769 PMC5878369 29549072

[72] Ellis LA Augustsson H Grødahl AI Pomare C Churruca K Long JC Implementation of e-mental health for depression and anxiety: a critical scoping review. J Community Psychol. (2020) 48(3):904–20. 10.1002/jcop.22309 31944324

[73] Powell BJ Waltz TJ Chinman MJ Damschroder LJ Smith JL Matthieu MM A refined compilation of implementation strategies: results from the expert recommendations for implementing change (ERIC) project. Implement Sci. (2015) 10(1):1–14. 10.1186/s13012-015-0209-1 25889199 PMC4328074

[74] Westfall JM Mold J Fagnan L. Practice-based research—“blue highways” on the NIH roadmap. J Am Med Assoc. (2007) 297(4):403–6. 10.1001/jama.297.4.403 17244837

