# Supplementary material for: Using the Consolidated Framework for Implementation Research to evaluate a nationwide depression prevention project (ImplementIT) from the perspective of health care workers and implementers: Results on the implementation of digital interventions for farmers
Source: Front Digit Health. 2023 Jan 23;4:1083143. doi: 10.3389/fdgth.2022.1083143 (PMC9907445; doi:10.3389/fdgth.2022.1083143)
Supplement: Supplementary file 2 [file Table2.docx]

**Supplementary material 2: Open questions and interview guide**

Open questions regarding barriers and facilitating factors in the implementation among staff involved in the referral process (asked every 6 months):

1. “*What difficulties did you encounter in the consultation and referral of the internet and tele-based health services?*”
2. “*What is going particularly well in terms of consultation and referral of the internet and tele-based health services?”*

Open questions regarding barriers and facilitating factors in the implementation among the implementation team (asked every 6 months):

1. “*Have you experienced any obstacles / difficulties / risks in implementing the internet- and tele-based health offerings in the last six months?*
2. *“Were there any hurdles that arose specifically in implementing the IBIs or specifically in implementing the TC?*”
3. “*What are supporting factors in the implementation process or what is helpful for you in implementing implementation activities?*”
