# Supplementary material for: Using the Consolidated Framework for Implementation Research to evaluate a nationwide depression prevention project (ImplementIT) from the perspective of health care workers and implementers: Results on the implementation of digital interventions for farmers
Source: Front Digit Health. 2023 Jan 23;4:1083143. doi: 10.3389/fdgth.2022.1083143 (PMC9907445; doi:10.3389/fdgth.2022.1083143)
Supplement: Supplementary file 3 [file Table3.docx]

**Supplementary Table.** Barriers and facilitating factors in counseling on digital prevention services at the CFIR dimension level among field workers and in-house staff (n = 62) and call center staff (n = 9).

| **Dimension** | | **Participants** | | | | | | | | | | **Definition** | **Example** |
| --- | --- | --- | --- | --- | --- | --- | --- | --- | --- | --- | --- | --- | --- |
|  | | Field staff  (N=62) | | | | Telecenter staff (N=9)) | | | All (N=71) | | |  |  |
|  | | **N** | | **%** | **K** | **N** | **%** | **K** | **N** | **%** | **K** |  |  |
| **I. INTERVENTION CHARACTERISTICS** | | | | | | | | | | | | | |
| ***Faciliators (n=5)*** | | | | | | | | | | | | | |
| \| Flexible use \| *2* \| *3* \| *2* \| *3* \| *33* \| *3* \| *5* \| *7* \| *5* \| It is perceived as an advantage that the health offers can be used flexibly in terms of time. \| *"Great interest in online offerings because time can be self-scheduled." (MS043)* \| \| --- \| --- \| --- \| --- \| --- \| --- \| --- \| --- \| --- \| --- \| --- \| --- \| \| Offer of a crisis hotline \| *4* \| *7* \| *5* \| *1* \| *11* \| *2* \| *5* \| *7* \| *7* \| Staff report that the intervention crisis hotline is helpful to insureds. \| *"Crisis hotline is great, for all insureds, straightforward" (MS099)* \| \| Anonymity \| *3* \| *5* \| *4* \| *1* \| *11* \| *1* \| *4* \| *6* \| *5* \| Employees see it as an advantage that the digital prevention services can be used anonymously. \| *"If someone is then seriously interested, they are glad that there is there is something like that. They don't have to go out of the house, the partner doesn't find out and neither does the neighborhood, the interested parties like above all the anonymity" (MS042)".* \| \| Location independence \| *3* \| *5* \| *3* \| *1* \| *11* \| *1* \| *4* \| *6* \| *4* \| Employees see it as an advantage that digital prevention services can be used  regardless of location. \| *"Basically, you notice that there is a need and interest, also because it is anonymous and you don't have to see a doctor or anything like that and you can do it from home." (MS126)* \| \| Variety of health offers \| *2* \| *3* \| *2* \| *1* \| *11* \| *1* \| *3* \| *4* \| *3* \| Employees particularly appreciate the variety of offerings. \| *Offer [is] large and broad. Something for everyone. (MS058)* \| | | | | | | | | | | | | | |
| **II. OUTER SETTING** | | | | | | | | | | | | | |
| ***Faciliators (n=5)*** | | | | | | | | | | | | | |
| \| Acceptance of mental health services/mental stress \| *13* \| *21* \| *15* \| *3* \| *33* \| *5* \| *16* \| *23* \| *20* \| According to employees, the insured accept the offer and appreciate the opportunities around mental health \| *"It is predominantly perceived as positive that something like this exists. Topics are seen as relevant by the insured!" (MS047)"* \| \| --- \| --- \| --- \| --- \| --- \| --- \| --- \| --- \| --- \| --- \| --- \| --- \| \| Interest in digital interventions \| *6* \| *10* \| *7* \| *4* \| *44* \| *6* \| *10* \| *14* \| *13* \| According to employees, the insured show great interest in the digital offerings. \| *"Insureds are often very pleased that there are online offerings." (MS043)* \| \| Quick contact with the insured \| *6* \| *10* \| *6* \| */* \| */* \| */* \| *6* \| *8* \| *6* \| The insureds seem to appreciate the quick and uncomplicated contact with the employees. \| *"Contact persons are there and help immediately. No waiting times like then let's make an appointment for in half a year." (MS089)* \| \| Prior knowledge of the insured about offers \| *1* \| *2* \| *1* \| *2* \| *22* \| *5* \| *3* \| *4* \| *6* \| It is helpful for the consultation from the employee's point of view if the insured already has prior knowledge of the health services. \| *"Often callers read about these offers in advance.*  *Then you can explain in more detail and pictures on the phone." (MS027)* \| \| Easy access to digital health services \| *2* \| *3* \| *2* \| */* \| */* \| */* \| *2* \| *3* \| *2* \| According to employees, insureds appreciate the uncomplicated access to services, such as quick appointment scheduling. \| *"Ease of access for insureds." (MS008)* \| | | | | | | | | | | | | | |
| ***Barriers (n=9)*** | | | | | | | | | | | | | |
|  | Inhibitions about using the internet/PC | *11* | | *18* | *12* | *5* | *56* | *12* | *16* | *23* | *24* | According to employees, the insured have inhibitions about using the internet or the computer. | *"Isolated reluctance of insured to engage in Internet activities, Increased among older persons." (MS026)* |
|  | Lack of time among the insured | *14* | | *23* | *15* | *1* | *11* | *1* | *15* | *21* | *16* | Staff report that insureds do not have time for counseling or to participate in health services. | *"Farmers rarely take the time to do it, it has to go that way." (MS128)* |
|  | Skepticism about offers for mental health issues/mental stress | *9* | | *15* | *10* | *3* | *33* | *5* | *12* | *17* | *15* | According to the employees, the insured persons have doubts about offers for mental health issues or mental stress. | *"Difficult topic and some rejection." (MS052)* |
|  | Prioritization of other tasks/topics | *9* | | *15* | *9* | */* | */* | */* | *9* | *13* | *9* | According to employees, the insured perceive other issues/problems as a priority and are therefore not interested in counseling or participating in the offerings. | *"Farmers are too busy doing other things." (MS049)* |
|  | Lack of problem comprehension | *6* | | *10* | *6* | *1* | *11* | *1* | *7* | *10* | *7* | According to the employees, there is a lack of understanding of problems with regard to mental health issues among the insured. | *"The offer of help often cannot be accepted because those affected either do not (want to) recognize the problem or the suffering pressure is not yet great enough." (MS058)* |
|  | Poor internet connection/missing PC | *4* | | *7* | *6* | *3* | *33* | *3* | *7* | *10* | *9* | From the perspective of employees, the internet connection at the insured is poor or there is no computer available for the use of online training. | *"Our older customers often don't have access to the Internet." (MS044)* |
|  | Lack of access authorization/insured status | *3* | | *5* | *6* | *2* | *22* | *4* | *5* | *7* | *10* | According to staff, some of the insured do not meet the prescribed requirements (insurance requirements, use of psychotherapy, etc.). | *"The majority of participants from the nursing tandem did not meet the insurance eligibility requirements." (MS041)* |
|  | Inhibitions of the insured during registration | *3* | | *5* | *4* | *1* | *11* | *1* | *4* | *6* | *5* | From the perspective of the employees, the insured are basically interested in the offers, but are reluctant to actually sign up. | *"Reluctance to take the final step and sign up. Basic interest [is] there, but then operational or family concerns are always put forward as to why it can't be done." (MS058)* |
|  | Forgetting about health offers by the insured | *1* | | *2* | *2* | */* | */* | */* | *1* | *1* | *2* | Employees report that insureds do not take advantage of health services because they forget they exist after the consultation. | *"The insured forget about the offers. Even though you know it's been addressed. Probably related to selective hearing" (MS042).* |
| **III. INNER SETTING** | | | | | | | | | | | | | |
| ***Faciliators (n=2)*** | | | | | | | | | | | | | |
| \| Appropriate materials for consultation \| *11* \| *18* \| *13* \| *1* \| *11* \| *1* \| *12* \| *17* \| *14* \| Informational materials such as flyers or overview lists make consultations for employees easier. \| *"With the help of the info sheets, a brief information of the insured is well feasible." (MS102)* \| \| --- \| --- \| --- \| --- \| --- \| --- \| --- \| --- \| --- \| --- \| --- \| --- \| \| Good teamwork within the SVLFG \| *9* \| *15* \| *10* \| */* \| */* \| */* \| *9* \| *13* \| *10* \| Employees appreciate the uncomplicated and fast cooperation, especially between the call center and field workers/office staff. \| *"Forwarding to the call center [is done] without any problems and quick completion." (MS015)* \| | | | | | | | | | | | | | |
| ***Barriers (n=8)*** | | | | | | | | | | | | | |
| \| Need for further materials/adaptation of materials \| *12* \| *19* \| *16* \| *2* \| *22* \| *2* \| *14* \| *20* \| *18* \| Employees express a need for additional materials/media (e.g., videos). \| *"A nice simple flyer. Just one and not too much. [...] Not a separate flyer for each offer.... one loses the overview so." (MS056)* \| \| --- \| --- \| --- \| --- \| --- \| --- \| --- \| --- \| --- \| --- \| --- \| --- \| \| Need for training/  information \| *12* \| *19* \| *18* \| *1* \| *11* \| *3* \| *13* \| *18* \| *21* \| Employees need training and targeted education to have more knowledge and confidence during counseling. \| *"I, as a field worker would need to be retrained to even reintroduce the program to insureds." (MS016)* \| \| Lack of time during consultations \| *12* \| *19* \| *20* \| */* \| */* \| */* \| *12* \| *17* \| *20* \| Staff members lack time during counseling to advise on internet- and tele-based services. \| *"Time issues to even consult on […] [health services]. Other issues/focuses/preferences are also still there." (MS019)* \| \| Lack of involvement in current developments of the project \| *10* \| *16* \| *13* \| *2* \| *22* \| *3* \| *12* \| *17* \| *16* \| Employees lack information about current campaign developments. \| *"Brief interim info, e.g., via WebEx, on recent changes or enhancements to offerings [would be helpful]." (MS011)* \| \| Bureaucratic burden \| */* \| */* \| */* \| *5* \| *56* \| *10* \| *5* \| *7* \| *10* \| Employees report problems during the referral process, such as completing the  consultation documentation form. \| *"Also, to really get all the information for the entry form. I'm thinking of the questions about whether seasonal workers or outside workers are involved in the operation. I have a hard time asking that on the side." (MS027)* \| \| Lack of support for employees from supervisors \| *3* \| *5* \| *4* \| */* \| */* \| */* \| *3* \| *4* \| *4* \| Employees feel inadequately supported by their supervisors. \| *„Supervisors share, but have problems with acceptance - in the management line” (MS081)* \| \| Problems during the referral process \| */* \| */* \| */* \| *3* \| *33* \| *4* \| *3* \| *4* \| *4* \| Employees report problems during the referral process of insureds. \| *“Ease of the referral process, too much pressed into scheme” (MS029)* \| \| Technical problems during consultation \| *1* \| *2* \| *2* \| */* \| */* \| */* \| *1* \| *1* \| *2* \| Technical difficulties (e.g. problems with the telephone system) make consultation difficult. \| *"The in-house telephone system is a disaster. Employees simply can't be reached." (MS108)* \|   **IV. CHARACTERISTICS OF INDIVIDUALS** | | | | | | | | | | | | | |
| ***Faciliators (n=1)*** | | | | | | | | | | | | | |
| \| Personal consultations \| *8* \| *13* \| *8* \| *2* \| *22* \| *2* \| *10* \| *14* \| *10* \| The employees consider the personal consultation of the insured persons to be important. \| " [...] Some then thaw out during the conversation and open up. " (MS025) \| \| --- \| --- \| --- \| --- \| --- \| --- \| --- \| --- \| --- \| --- \| --- \| --- \| | | | | | | | | | | | | | |
| ***Barriers (n=6)*** | | | | | | | | | | | | | |
|  | COVID-19 related reduction in onsite contacts with insured persons | *12* | | *19* | *14* | *1* | *11* | *1* | *13* | *18* | *15* | Due to the pandemic, only telephone consultations can take place, which means that personal confidential contact is lost. | *"Since there were hardly any business trips at the moment due to Covid-19 (or none at all in the period from March to July) there has also been no personal contact with the injured outside of phone calls, so it has been more difficult to assess their mood/psychological condition." (MS100)* |
|  | Addressing mental health issues | *10* | | *16* | *11* | *1* | *11* | *1* | *11* | *16* | *12* | Employees find it difficult to address mental health issues. | *"Counseling someone who you think may have mental health problems is a very difficult approach for me. Since I'm not usually a trusted person and I'm not a psychologist, it's hard for me." (MS047)* |
|  | Little routine in advising on health care services | *5* | | *8* | *5* | *4* | *44* | *9* | *9* | *13* | *14* | The employees lack routine and confidence in consulting. | *"All available, but little routine in it." (MS019)* |
|  | Difficulties in recognizing the relevance of mental health services | *6* | | *10* | *8* | *2* | *22* | *4* | *8* | *11* | *12* | The employees have problems recognizing whether or which health offer is appropriate for the respective insured person. | *"To identify whether the issue is necessary in the operation." (MS021)* |
|  | Lack of consultations | *4* | | *7* | *5* | *2* | *22* | *2* | *6* | *9* | *7* | Staff report having done little or no consultations on internet- and tele-based health services in recent months. | *"I have not consulted on any of the above offerings in the last six months." (MS019)* |
|  | No time for self-experience through employee participation | *1* | | *2* | *2* | */* | */* | */* | *1* | *1* | *2* | Employees lack the time to participate in a health program themselves and thus get to know it better. | *"Time to take a closer look at the issue yourself and go through a proposal like this yourself." (MS057)* |
| **V. IMPLEMENTATION PROCESS** | | | | | | | | | | | | | |
| ***Faciliators (n=1)*** | | | | | | | | | | | | | |
|  | Increased advertising of the digital health service in the media | *2* | | *3* | *4* | *1* | *11* | *1* | *3* | *4* | *5* | Employees see the increased media presence of digital health offerings as helpful. | *"Promote [health offerings] more in the media." (M052)* |
| ***Barriers (n=1)*** | |  | | | | | | | | | | | |
| Lack of clarity of the SVLFG website | | | *5* | *8* | *5* | */* | */* | */* | *5* | *7* | *5* | Employees describe the SVLFG homepage as confusing and it is difficult for insureds to find their way around. | *"Insureds report back that they have a hard time finding their way around the website and take a long time to find the offerings." (MS092)* |
